# Taxonomic study of the *Pinelemabailongensis* species group with descriptions of six new species from China (Araneae, Telemidae)

**DOI:** 10.3897/zookeys.784.27758

**Published:** 2018-09-12

**Authors:** Huifeng Zhao, Zhiyuan Yao, Yang Song, Shuqiang Li

**Affiliations:** 1 Institute of Zoology, Chinese Academy of Sciences, Beijing 100101, China Institute of Zoology, Chinese Academy of Sciences Beijing China; 2 College of Life Science, Shenyang Normal University, Shenyang, Liaoning 110034, China Shenyang Normal University Shenyang China

**Keywords:** Haplogynae, new combination, spider, *
Telema
*

## Abstract

The *Pinelemabailongensis* Wang & Li, 2012 species group of the spider family Telemidae Fage, 1913 from Guangxi and Guizhou, China is revised. Previously, this species group contained two species: *P.bailongensis* and *P.xiushuiensis* Wang & Li, 2016. In this study, four species are transferred from *Telema* Simon, 1882 to *Pinelema* Wang & Li, 2012, and treated as members of the *P.bailongensis* species group. They are *P.cordata* (Wang & Li, 2010) **comb. n.**, *P.liangxi* (Zhu & Chen, 2002) **comb. n.**, *P.strentarsi* (Lin & Li, 2010) **comb. n.** and *P.zhewang* (Chen & Zhu, 2009) **comb. n.** Additionally, six new species belonging to the species group are described: *P.cheni* Zhao & Li, **sp. n.** (♂♀), *P.huoyan* Zhao & Li, **sp. n.** (♂♀), *P.lizhuang* Zhao & Li, **sp. n.** (♂♀), *P.wangshang* Zhao & Li, **sp. n.** (♂♀), *P.wenyang* Zhao & Li, **sp. n.** (♂♀) and *P.yunchuni* Zhao & Li, **sp. n.** (♂♀). A key to males is provided.

## Introduction

Telemidae Fage, 1913 currently contains 79 species in ten genera worldwide (World Spider Catalog 2018). It has fragmented distributions in rainforest or karst caves of tropical Africa, Eurasia, and the New World (Song et al. 2017a). *Pinelema* Wang & Li, 2012, the second largest genus of the family, occuring in karst caves or leaf litter. A total of 15 *Pinelema* species was known from China and Vietnam before the current study.

The *P.bailongensis* species group, characterized by a distinctly long embolus relative to the bulb, currently contains only two species: *P.bailongensis* Wang & Li, 2012 and *P.xiushuiensis* Wang & Li, 2016. The species group is restricted to karst caves in southern Guizhou and western Guangxi, China. In this paper, four species are transferred from *Telema* Simon, 1882 to *Pinelema*, and six new species are described.

## Material and methods

The individuals of *Pinelemabailongensis* species group studied here are from the cave expeditions in southern China during last decade.

All specimens were examined and measured using a LEICA M205 C stereomicroscope. All measurements are given in millimeters. Leg measurements are as follows: total length (femur, patella, tibia, metatarsus, tarsus). The habitus, left male palp, and receptacle were photographed using an Olympus C7070 digital camera. Female genitalia were removed and treated in lactic acid before being photographed. Images were combined using Helicon Focus image stacking software. For SEM images, the left male palp was photographed using a Hitachi SU8010 Environmental Scanning Electron Microscope.

To confirm the stable morphology of male palps for each species in the *P.bailongensis* species group, photos of additional individuals are provided in the supplementary material. Genetic distances were obtained for four to five individuals of each species. Genomic DNA was extracted from the prosomas of females. Standard barcode COI (650 bp) was amplified using the primer pair LCO1490 (5’-GGTCAACAAATCATAAAGATATTGG-3’) and HCO2198 (5’-TAAACTTCAGGGTGACCAAAAAATCA-3’) (Folmer et al. 1994). The PCR protocol consisted of initial denaturing of 95 °C for 5 min, 5 cycles of 95 °C for 30 s, 45 °C for 30 s, and 72 °C for 30 s, then 35 cycles of 95 °C for 30 s, 51 °C for 30 s, and 72 °C for 30 s, with a final extension of 72 °C for 5 min. All PCR positive products were purified and sequenced by Tianyihuiyuan Biotech Co., Ltd (Beijing, China) using an ABI 3730 automated sequencer. Raw ABI sequences were edited by hand in BioEdit (Hall 1999). Uncorrected pairwise distances between species in the *P.bailongensis* species group were calculated using MEGA 5.0 (Tamura et al. 2011). All sequences are deposited in GenBank, and the accession numbers are listed in Table 1.

**Table 1.** GenBank accession numbers and mean inter-specific uncorrected p-distances of species in the *P.bailongensis* species group from COI data.

	Species	GenBank accession number	1	2	3	4	5	6	7	8	9	10	11
1	* P. bailongensis *	MH643817–MH643821											
2	* P. cheni *	MH643822–MH643825	0.169										
3	* P. cordata *	MH643826–MH643830	0.151	0.149									
4	* P. huoyan *	MH643831–MH643835	0.168	0.163	0.164								
5	* P. liangxi *	MH643836–MH643840	0.146	0.161	0.154	0.155							
6	* P. lizhuang *	MH643841–MH643844	0.151	0.169	0.149	0.177	0.153						
7	* P. strentarsi *	MH643845–MH643849	0.161	0.164	0.158	0.154	0.170	0.165					
8	* P. wangshang *	MH643850–MH643853	0.160	0.166	0.159	0.154	0.151	0.162	0.158				
9	* P. wenyang *	MH643854–MH643858	0.156	0.167	0.160	0.154	0.177	0.159	0.169	0.162			
10	* P. xiushuiensis *	MH643859–MH643863	0.137	0.146	0.169	0.149	0.134	0.154	0.139	0.166	0.162		
11	* P. yunchuni *	MH643864–MH643867	0.169	0.147	0.171	0.133	0.145	0.164	0.135	0.153	0.150	0.146	
12	* P. zhewang *	MH643868–MH643872	0.142	0.155	0.122	0.154	0.143	0.152	0.148	0.157	0.154	0.146	0.128

**Figure 1.**
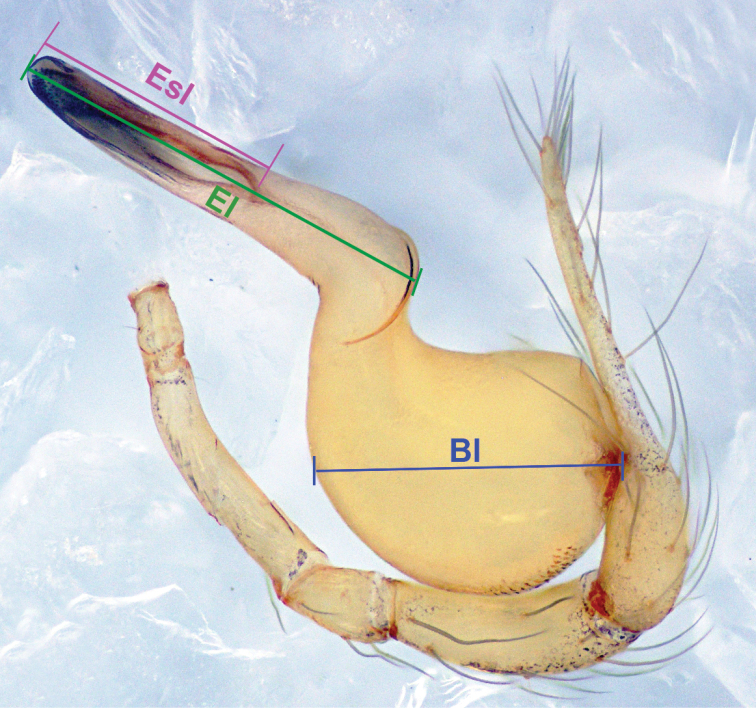
*Pinelemabailongensis*, male palp, retrolateral view showing bulb length (Bl), embolus length (El) and embolic slit length (Esl).

References to figures in the cited papers are listed in lowercase (figure or figs); figures from this paper are noted with an initial capital (Figure or Figs). The following abbreviations are used in the text or figures:

**Bl** Bulb length, the bulbal bisector line from the junction of the bulb and cymbium to the distal ridge of bulb (blue line in Figure 1).

**Ca** Cymbial apophysis.

**El** Embolus length, the bisector line of the embolus from base to tip (green line in Figure 1).

**El/Bl** ratio of El to Bl. The gap between the El/Bl ratio ranges between two species should be larger or equal to the El/Bl ratio range within each species.

**Em** Embolus.

**Es** Embolic slit.

**Esl**Es length (purple line in Figure 1).

**Esl/El** The ratio of the Esl and El. The gap between the Esl/El ratio ranges between two species should be larger or equal to the Esl/El ratio range within each species.

**Pa** Papillae on bulb proximo-retrolaterally.

**Re** Receptacle.

**Sr** Spiral ridge of embolus.

Abbreviations of institutes:

**IZCAS**Institute of Zoology, Chinese Academy of Sciences, Beijing, China.

**MHBU** Museum of Hebei University, Baoding, China.

**MLR** Maolan National Natural Reserve, Libo, Guizhou, China.

## Taxonomy

### Family Telemidae Fage, 1913

#### Genus Pinelema

Taxon classificationAnimaliaAraneaeTelemidae

Wang & Li, 2012

##### Type species.

*Pinelemabailongensis* Wang & Li, 2012 from Guangxi, China.

##### Composition.

The total number of *Pinelema* species has increased to 25. They are *P.bailongensis* Wang & Li, 2012, *P.cheni* Zhao & Li, sp. n., *P.cordata* (Wang & Li, 2010) comb. n., *P.cunfengensis* Zhao & Li, 2017, *P.curcici* Wang & Li, 2016, *P.damtaoensis* Zhao & Li, 2018, *P.huobaensis* Wang & Li, 2016, *P.huoyan* Zhao & Li, sp. n., *P.laensis* Zhao & Li, 2018, *P.liangxi* (Zhu & Chen, 2002) comb. n., *P.lizhuang* Zhao & Li, sp. n., *P.nuocnutensis* Zhao & Li, 2018, *P.pacchanensis* Zhao & Li, 2018, *P.podiensis* Zhao & Li, 2017, *P.qingfengensis* Zhao & Li, 2017, *P.spirulata* Zhao & Li, 2018, *P.strentarsi* (Lin & Li, 2010) comb. n., *P.wangshang* Zhao & Li, sp. n., *P.wenyang* Zhao & Li, sp. n., *P.xiezi* Zhao & Li, 2018, *P.xiushuiensis* Wang & Li, 2016, *P.yaosaensis* Wang & Li, 2016, *P.yunchuni* Zhao & Li, sp. n., *P.zhenzhuang* Zhao & Li, 2018, and *P.zhewang* (Chen & Zhu, 2009) comb. n.

##### Diagnosis and descriptions.

See Wang and Li (2016) and Zhao et al. (2018).

##### Distribution.

China (Guangxi, Guizhou, Yunnan), Vietnam (Vinh Phuc, Quang Binh, Phu Tho, Bac Kan).

#### Pinelema bailongensis species group

Taxon classificationAnimaliaAraneaeTelemidae

##### Diagnosis.

Species of *Pinelemabailongensis* species group can be distinguished from all other *Pinelema* species by embolus distinctly longer than bulb and by endogyne U-shaped, J-shaped or spiral. In contrast, embolus of other *Pinelema* species shorter than bulb and endogyne bag-like.

##### Distribution.

China (western Guangxi, southern Guizhou).

##### Comments.

*Pinelemabailongensis* species group is recognized as the first species group in *Pinelema* according to the long embolus relative to the bulb. Morphologically, the species in this group have quite simple and almost identical copulatory organs, only proportions of the bulb and its parts can help to separate the species, endogynes can not be used for distinguishing species (except *P.bailongensis* and *P.wangshang* Zhao & Li, sp. n.). Genetically, each species in this group owns very significant difference comparing the congeners (Table 1).

#### Illustrated key to males of the *Pinelemabailongensis* species group

**Table:** 

1	Eyes present (1.1)	**2**
–	Eyes absent (1.2)	**8**
	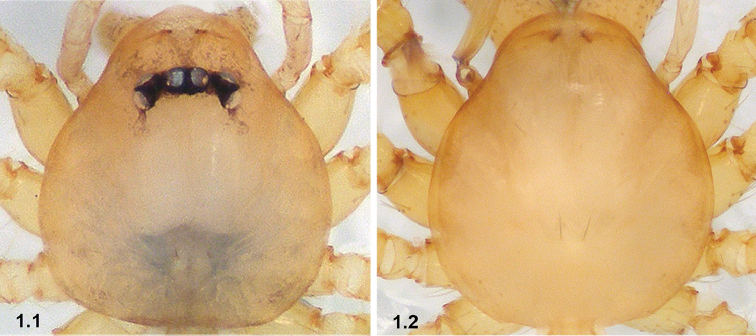	
2	Bulb without papillae proximo-retrolaterally (2.1)	**3**
–	Bulb with papillae proximo-retrolaterally (2.2)	**4**
	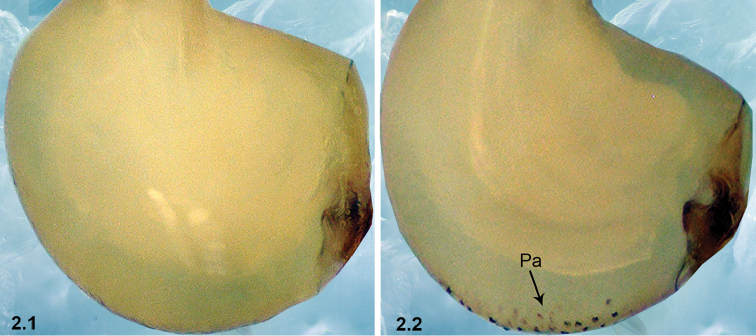	
3	Bulb curved dorso-distally (arrowed in 3.1a); Esl/El ratio: 0.51–0.55 (n = 5, mean: 0.53) (3.1b); El/Bl ratio: 1.47–1.54 (n = 5, mean: 1.49)	***P.wenyang* Zhao & Li, sp. n.**
–	Bulb curved dorso-medially (arrowed in 3.2a); Esl/El ratio: 0.63–0.67 (n = 6, mean: 0.65) (3.2b); El/Bl ratio: 1.24–1.31 (n = 6, mean: 1.27)	***P.lizhuang* Zhao & Li, sp. n.**
	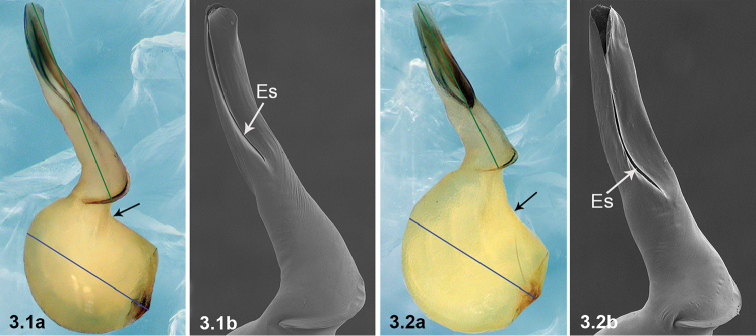	
4	Esl/El ratio: 0.51–0.62 (n = 10) (4.1)	**5**
–	Esl/El ratio: 0.33–0.45 (n = 14) (4.2)	**6**
	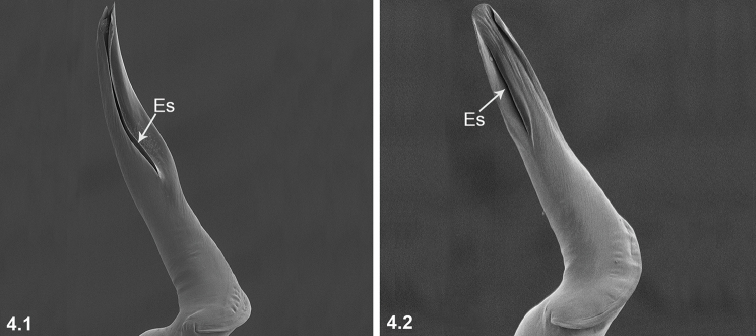	
5	Embolus with small apophyses distal-retrolaterally (arrowed in 5.1a); Esl/El ratio: 0.58–0.62 (n = 5, mean: 0.60) (5.1b)	***P.bailongensis* Wang & Li, 2012**
–	Embolus without small apophyses distal-retrolaterally (5.2a); Esl/El ratio: 0.51–0.54 (n = 5, mean: 0.52) (5.2b)	***P.cordata* (Wang & Li, 2010)**
	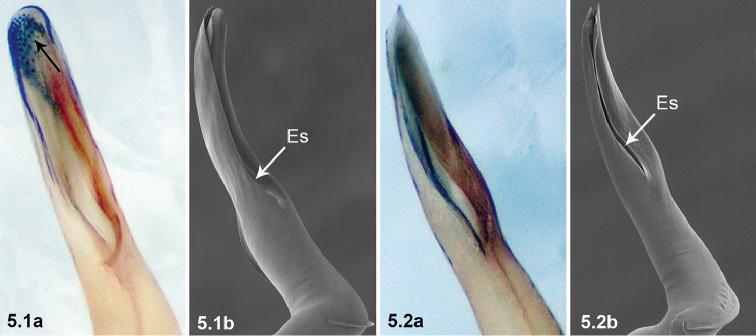	
6	Embolus with a bifurcated tip (arrowed in 6.1a); bulb with a right-angled bend dorso-subdistally (arrowed in 6.1b)	***P.cheni* Zhao & Li, sp. n.**
–	Embolus with a slightly arch-shaped tip (arrowed in 6.2a); bulb with an obtuse-angled bend dorso-distally (arrowed in 6.2b)	**7**
	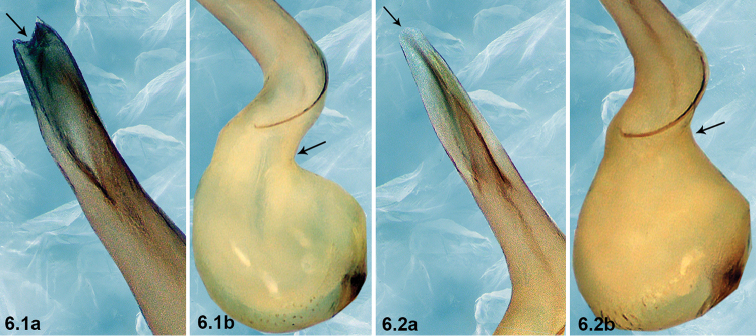	
7	Bulb kidney-shaped and curved dorso-medially (arrow in 7.1), El/Bl ratio 1.37–1.45 (n = 5, mean: 1.41) (7.1)	***P.wangshang* Zhao & Li, sp. n.**
–	Bulb pear-shaped and not curved dorso-medially (arrow in 7.2), El/Bl ratio 1.78–1.82 (n = 4, mean: 1.80) (7.2)	***P.yunchuni* Zhao & Li, sp. n.**
	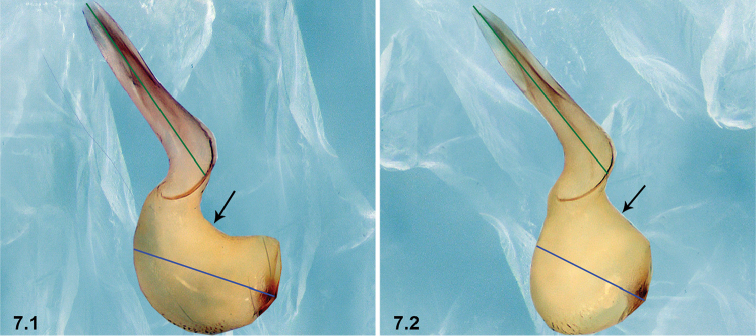	
8	Embolus with small apophyses distal-retrolaterally (arrowed in 8.1); Esl/El ratio 0.72–0.78 (n = 5, mean: 0.76)	***P.huoyan* Zhao & Li, sp. n.**
–	Embolus without small apophyses distal-retrolaterally (8.2); Esl/El ratio 0.48–0.63 (n = 18)	**9**
	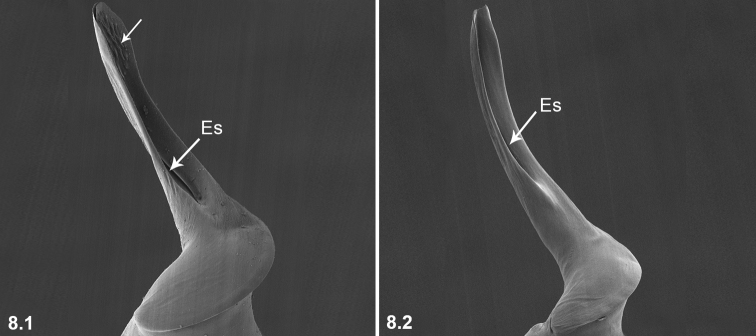	
9	El/Bl ratio: 1.58–1.83 (n = 9) (9.1)	**10**
–	El/Bl ratio: 1.14–1.25 (n = 9) (9.2)	**11**
	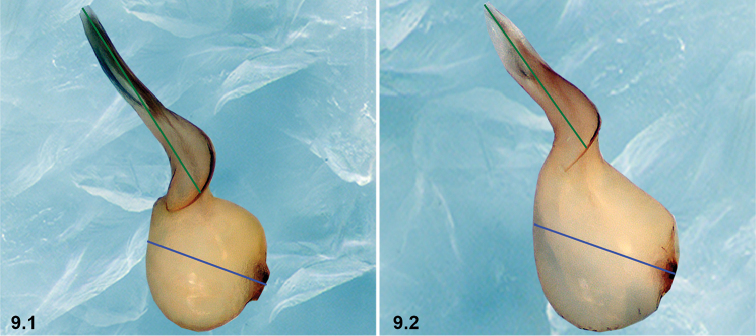	
10	Ca short (10.1a, scale bar 0.2 mm); Esl/El ratio: 058–0.63 (n = 4, mean 0.60) (10.1b)	***P.zhewang* (Chen & Zhu, 2009)**
–	Ca long (10.2a, scale bar 0.2 mm); Esl/El ratio: 050–0.52 (n = 5, mean 0.51) (10.2b)	***P.xiushuiensis* Wang** & **Li, 2016**
	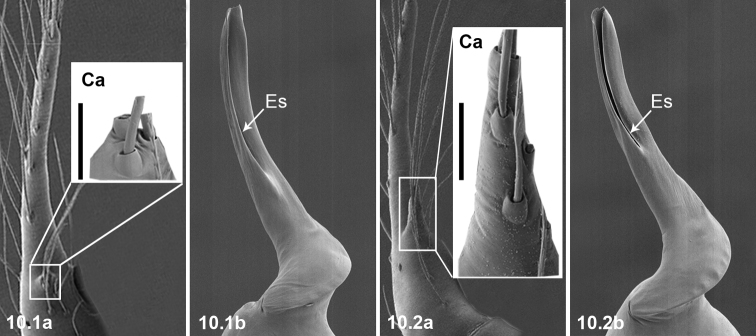	
11	Embolus straight (arrow 1 in 11.1); bulb protruding ventro-subdistally (arrow 2 in 11.1) and slightly curved dorso-medially (arrow 3 in 11.1)	***P.strentarsi* (Lin & Li, 2010)**
–	Embolus curved (arrow 1 in 11.2); bulb not protruding ventro-subdistally (arrow 2 in 11.2) and not curved dorso-medially (arrow 3 in 11.2)	***P.liangxi* (Zhu & Chen, 2002)**
	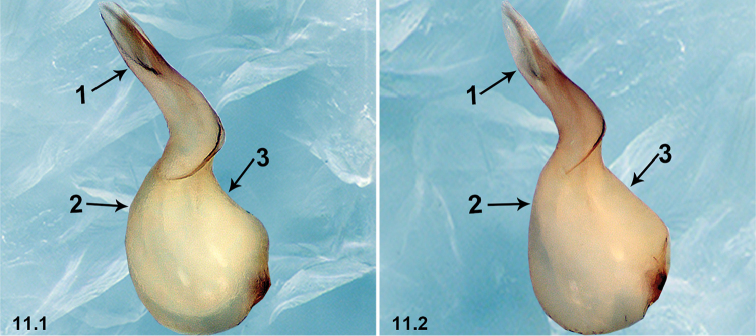	

#### Pinelema bailongensis

Taxon classificationAnimaliaAraneaeTelemidae

Wang & Li, 2012

Figs 1 , 31

Pinelema bailongensis : Wang and Li 2012: 82, figs 1–17 (♂♀); Song et al. 2017b: 85, figs 7A, 8A, 9A, 10A, 11A, 12A (♂).

##### Material examined.

Holotype ♂ (IZCAS): China, Guangxi Zhuang Autonomous Region, Baise Prefecture, Pingguo County, Bailong Cave, 23°19.094’N, 107°34.387’E, 111 m, 1.VIII.2009, C. Wang & Z. Yao leg. Paratypes (IZCAS): 1♂ and 4 ♀, same data as holotype.

##### Other material examined.

5♂ and 5♀ (molecular vouchers, IZCAS), same data as holotype.

##### Diagnosis.

*Pinelemabailongensis* resembles *P.cordata* (see Figure 5 and Wang and Li 2010: 9, figs 11–15) but can be distinguished by following characters: small apophyses present on embolus distal-retrolaterally (see Song et al. 2017b: figure 8A and Wang and Li 2012: figure 4) (apophyses absent on embolus distal-retrolaterally in *P.cordata*), larger Esl/El ratio (0.58–0.62, n = 5, mean: 0.60, Suppl. material 1: Figure S1) (smaller Esl/El ratio 0.51–0.54, n = 5, mean: 0.52 in *P.cordata*), and shape of receptacle spiral (see Wang and Li 2012: figs 8, 11) (receptacle U-shaped in *P.cordata*).

##### Description.

El/Bl ratio 1.40–1.48 (n=5, mean: 1.43, Suppl. material 1: Figure S1), Esl/El ratio 0.58–0.62 (n = 5, mean: 0.60, Suppl. material 1: Figure S1). For more detailed descriptions see Wang and Li (2012) and Song et al. (2017b).

##### Distribution.

China (Guangxi, Figure 31), known only from the type locality.

#### Pinelema cheni

Taxon classificationAnimaliaAraneaeTelemidae

Zhao & Li sp. n.

http://zoobank.org/909EE4D6-1A37-4BC2-A146-4280791CFED1

Figs 2 , 3 , 4 , 31

##### Type material.

**Holotype** ♂ (IZCAS): China, Guangxi Zhuang Autonomous Region, Hechi Prefecture, Du’an County, Gaoling Town, Jiangzhong Village, Huoyan Cave, 24°01.820'N, 108°04.720'E, 243 m, 11.II.2015, Y. Li and Z. Chen leg. **Paratypes** (IZCAS): 3♂ and 3♀, same data as holotype.

##### Other material examined.

4♀ (molecular vouchers, IZCAS), same data as holotype.

##### Etymology.

The specific name is a patronym in honor of the collector Zhigang Chen.

##### Diagnosis.

*Pinelemacheni* Zhao & Li, sp. n. can be easily distinguished from all other congeners by following characters: bifurcated tip of embolus (arrow 1 in Figure 3D) (arch-shaped tips on emboli in other congeners), right-angled bend onbulb dorso-subdistally (arrow 2 in Figure 3D) (no right-angled bend on bulbs in other congeners), and smaller Esl/El ratio (0.33–0.37, n = 5, mean: 0.34, Suppl. material 1: Figure S2) (larger Esl/El ratio 0.42–0.78 in other congeners).

**Figure 2.**
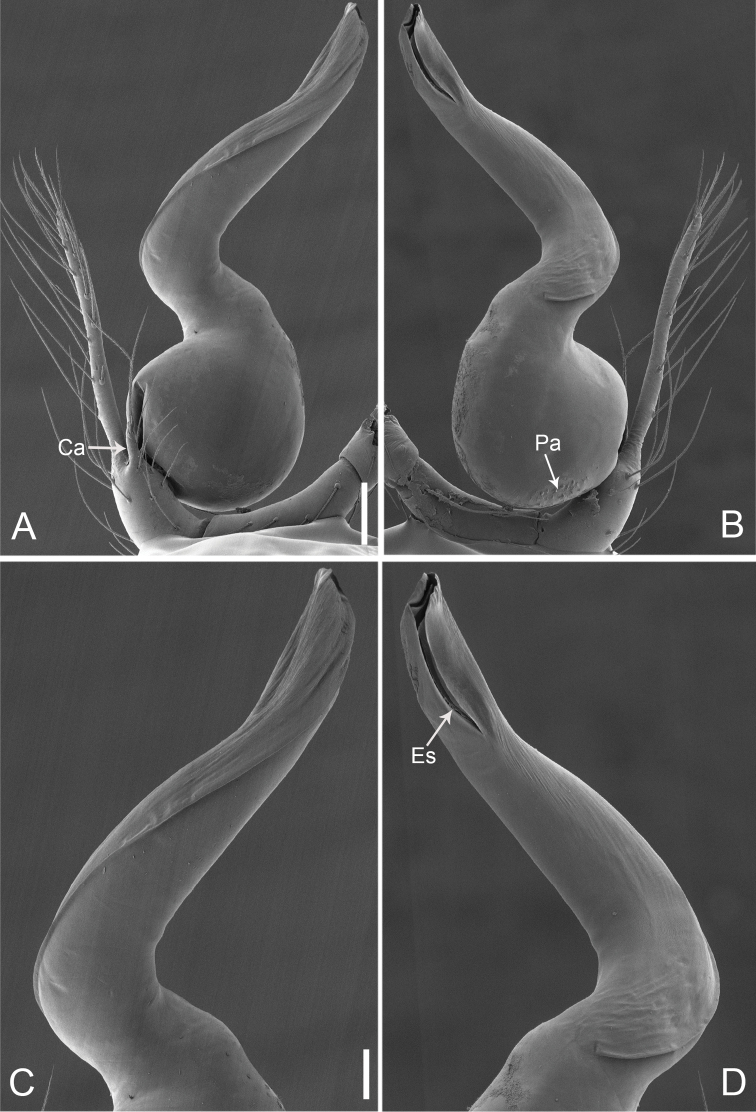
*Pinelemacheni* Zhao & Li, sp. n., male. **A** Palp, prolateral view **B** Palp, retrolateral view **C** Embolus, prolateral view **D** Embolus, retrolateral view. Scale bars: 0.1 mm (**A–B**), 0.05 mm (**C–D**).

**Figure 3.**
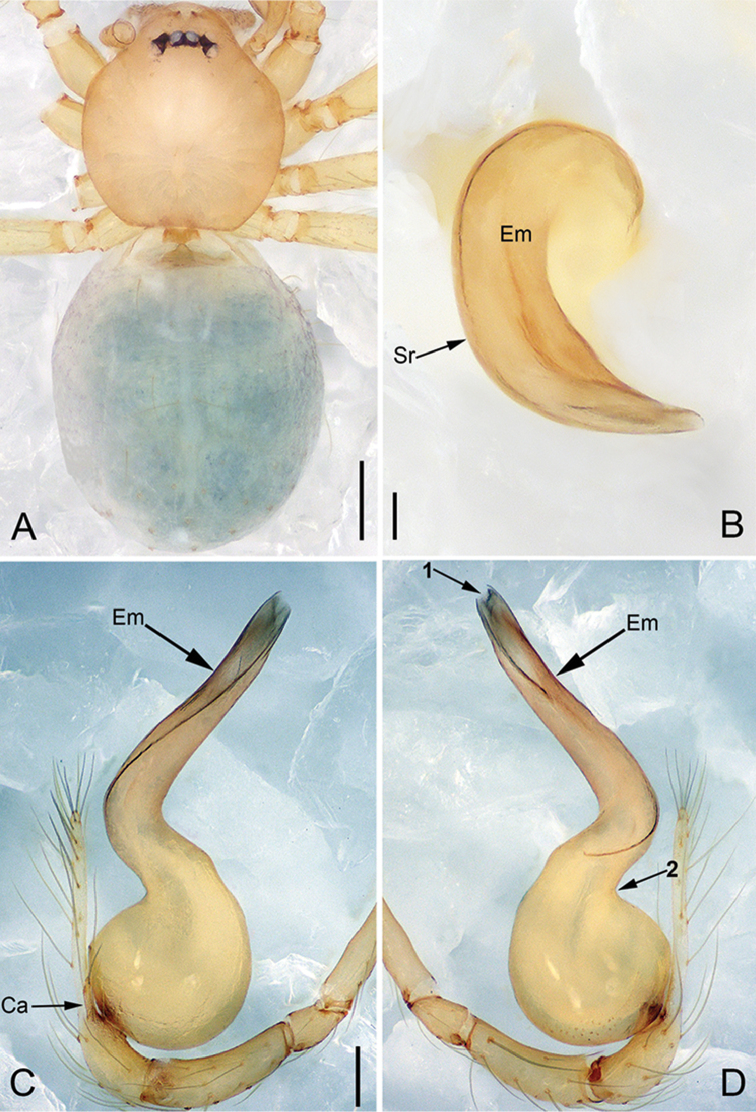
*Pinelemacheni* Zhao & Li, sp. n., male holotype. **A** Habitus, dorsal view **B** Embolus, apical view **C** Palp, prolateral view **D** Palp, retrolateral view. Scale bars: 0.2 mm (**A**), 0.05 mm (**B**), 0.1 mm (**C–D**).

##### Description.

**Male (holotype)**: Total length 1.38. Carapace 0.57 long, 0.51 wide. Abdomen 0.79 long, 0.69 wide. Carapace light brown (Figure 3A). Six eyes ringed with black, clypeus height 0.07, ocular quadrangle 0.17 wide (Figure 3A). Chelicerae, legs, labium, and endites yellow. Sternum light brown with sparse setae. Leg measurements: I 4.58 (1.36, 0.22, 1.48, 0.95, 0.57); II 3.80 (1.13, 0.22, 1.19, 0.75, 0.51); III 2.74 (0.81, 0.20, 0.79, 0.50, 0.44); IV 3.42 (1.06, 0.22, 0.97, 0.71, 0.46). Abdomen light blue with several long setae.

Palp: Tibia 2.8 times longer than patella, cymbium 2.0 times longer than tibia, cymbial apophysis cone-shaped (Figs 2A, 3C); bulb with papillae proximo-retrolaterally (Figure 2B) and a right-angled bend dorso-subdistally (arrow 2 in Figure 3D); embolus tube-shaped, with a bifurcated tip (arrow 1 in Figure 3D), spiral ridge dark brown (Figure 3B), El/Bl ratio 1.65 (Figure 3D), Esl/El ratio 0.37 (Figure 3D).

**Figure 4.**
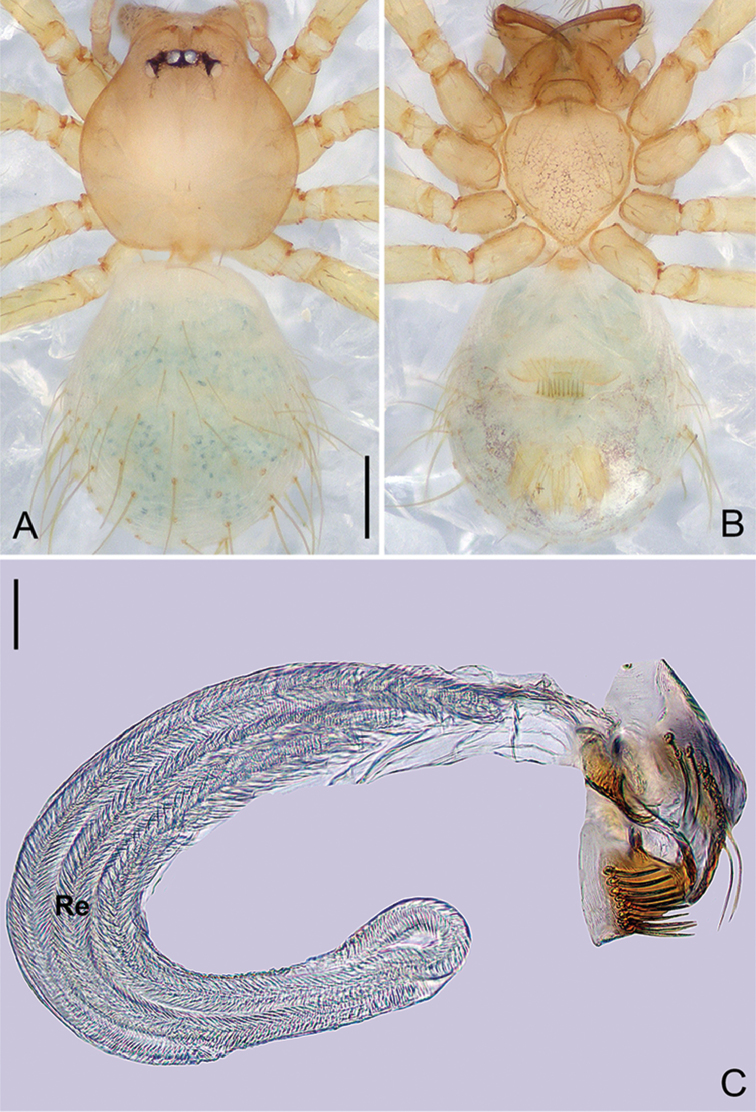
*Pinelemacheni* Zhao & Li, sp. n., female paratype. **A** Habitus, dorsal view **B** Habitus, ventral view **C** Endogyne, lateral view. Scale bars: 0.2 mm (**A–B**), 0.05 mm (**C**).

**Female**: Total length 1.30. Carapace 0.59 long, 0.54 wide. Abdomen 0.71 long, 0.59 wide. Coloration as in male and abdomen with many long setae (Figure 4A, B). Six eyes, well developed, clypeus height 0.10, ocular quadrangle 0.17 wide (Figure 4A). Leg measurements: I 4.38 (1.31, 0.21, 1.39, 0.86, 0.61); II 3.72 (1.13, 0.21, 1.13, 0.71, 0.54); III 2.73 (0.84, 0.19, 0.78, 0.48, 0.44); IV 3.28(1.03, 0.21, 0.96, 0.62, 0.46). Insemination duct thinner than receptacle (Figure 4C); receptacle with multiple membranous tubes, U-shaped (Figure 4C).

##### Variation.

In 4♂ paratypes: El/Bl ratio 1.69–1.78, Esl/El ratio 0.33–0.36.

##### Distribution.

China (Guangxi, Figure 31), known only from the type locality.

#### Pinelema cordata

Taxon classificationAnimaliaAraneaeTelemidae

(Wang & Li, 2010) comb. n.

Figs 5 , 31

Telema cordata : Wang and Li 2010: 9, figs 11–15 (♂♀).

##### Material examined.

Holotype ♂ (IZCAS): China, Guangxi Zhuang Autonomous Region, Nanning Prefecture, Wuming County, Yiling Cave, 23°02.374'N, 108°17.529'E, 151 m, T: 23 °C, RH: 86%, 31.VII.2009, C. Wang and Z. Yao leg. Paratypes (IZCAS): 1♂ and 4♀ (IZCAS), same data as holotype.

##### Other material examined.

5♂ and 5♀ (molecular vouchers, IZCAS), same data as holotype.

##### Diagnosis.

**Figure 5.**
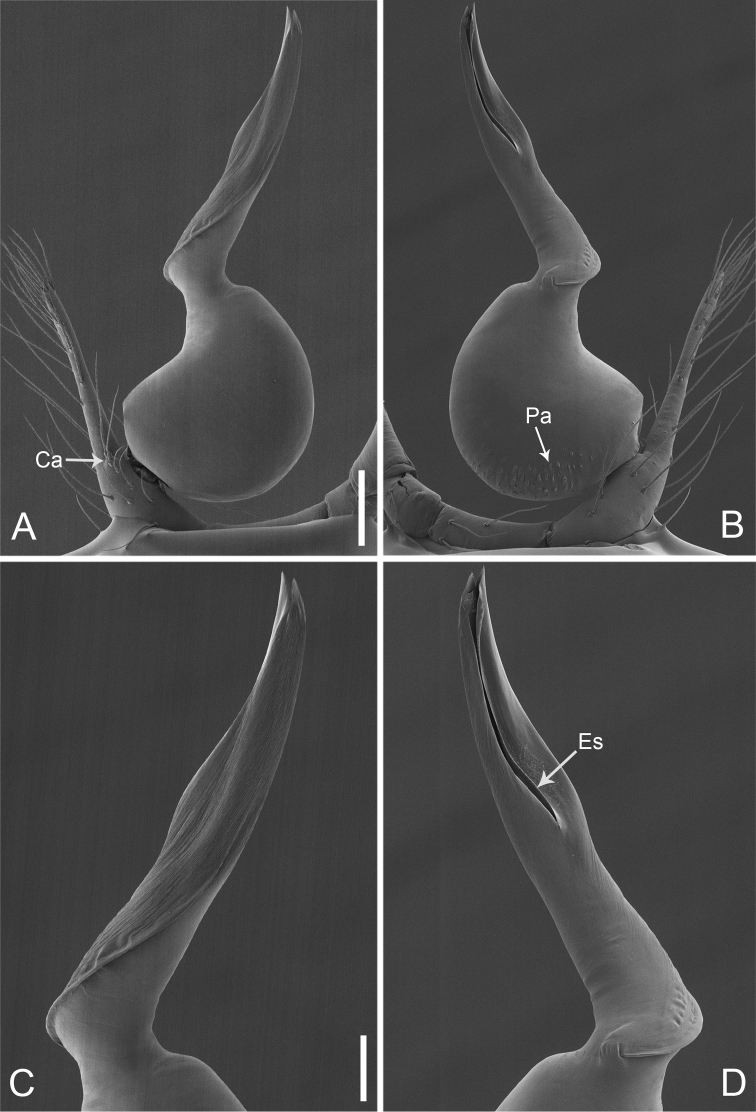
*Pinelemacordata* comb. n., male. **A** Palp, prolateral view **B** Palp, retrolateral view **C** Embolus, prolateral view **D** Embolus, retrolateral view. Scale bars: 0.1 mm (**A–B**), 0.05 mm (**C–D**).

This species resembles *P.bailongensis* (see Figure 1, Wang and Li 2012: 82, figs 1–17 and Song et al. 2017b: 85, figs 7A, 8A, 9A, 10A, 11A, 12A) but can be distinguished by following characters: small apophyses absent on embolus distal-retrolaterally (Figure 5D) (small apophyses present on embolus distal-retrolaterally in *P.bailongensis*), smaller Esl/El ratio (0.51–0.54, n = 5, mean: 0.53, Suppl. material 1: Figure S3) (larger Esl/El ratio 0.58–0.62, n = 5, mean: 0.60 in *P.bailongensis*), and receptacle U-shaped (see Wang and Li 2010: figure 13C) (receptacle spiral in *P.bailongensis*).

##### Description.

Male palp: Bulb with papillae proximo-retrolaterally (Figure 5B); El/Bl ratio 1.51–1.54 (n = 5, mean: 1.52, Suppl. material 1: Figure S3), Esl/El ratio 0.51–0.54 (n = 5, mean: 0.52, Suppl. material 1: Figure S3). For more detailed descriptions, see Wang and Li (2010).

##### Comments.

This species is transferred to *Pinelema* because it shares similar morphological characters with *P.bailongensis*, such as the long, tube-shaped embolus (see Figure 5 and Wang and Li 2010: figs 11A–B, 12B), the presence of a distinct cymbial apophysis in the male palp prolaterally (see Figure 5A and Wang and Li 2010: figure 11B), and the U-shaped and medially strongly curved receptacle (see Wang and Li 2010: figure 13).

##### Distribution.

China (Guangxi, Figure 31), known only from the type locality.

#### Pinelema huoyan

Taxon classificationAnimaliaAraneaeTelemidae

Zhao & Li sp. n.

http://zoobank.org/FED63C61-C96B-460D-A338-8B2C142EE4B8

Figs 6 , 7 , 8 , 31

##### Type material.

**Holotype** ♂ (IZCAS): China, Guangxi Zhuang Autonomous Region, Hechi Prefecture, Du’an County, Gaoling Town, Jiangzhong Village, Huoyan Cave, 24°01.820'N, 108°04.720'E, 243 m, 11.II.2015, Y. Li and Z. Chen leg. **Paratypes** (IZCAS): 4♂ and 6♀, same data as holotype.

##### Other material examined.

5♀ (molecular vouchers, IZCAS), same data as holotype.

##### Etymology.

The species epithet refers to the type locality; noun.

##### Diagnosis.

**Figure 6.**
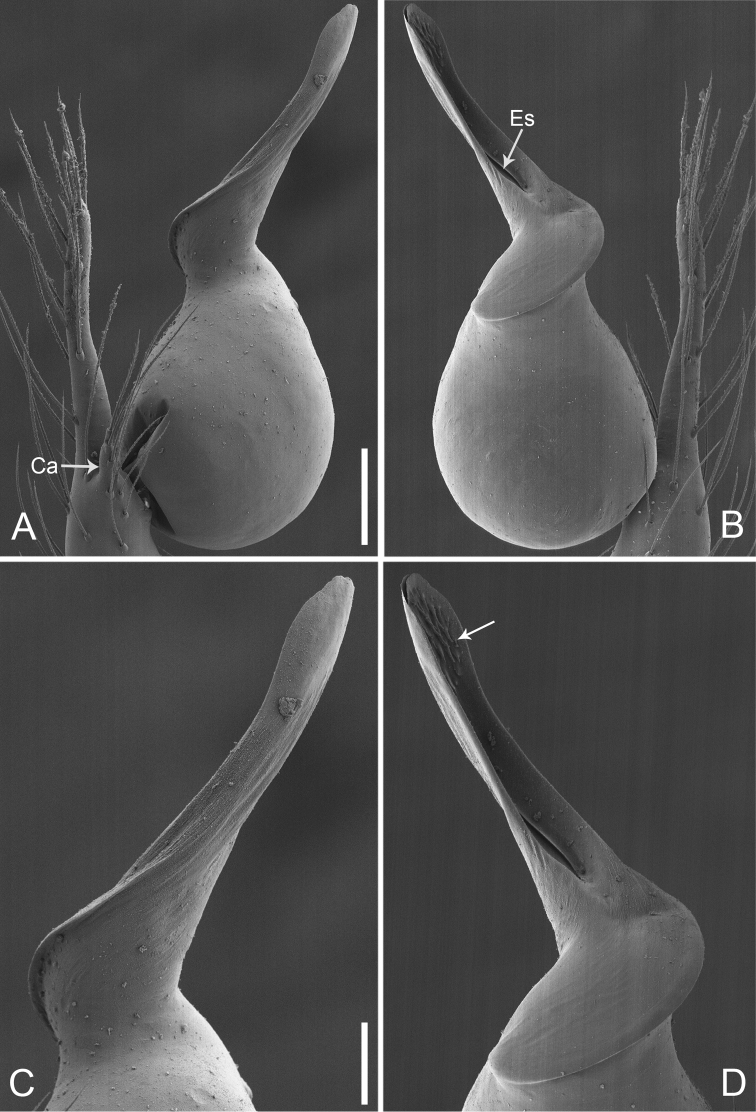
*Pinelemahuoyan* Zhao & Li, sp. n., male. **A** Palp, prolateral view **B** Palp, retrolateral view **C** Embolus, prolateral view **D** Embolus, retrolateral view. Scale bars: 0.1 mm (**A–B**), 0.05 mm (**C–D**).

**Figure 7.**
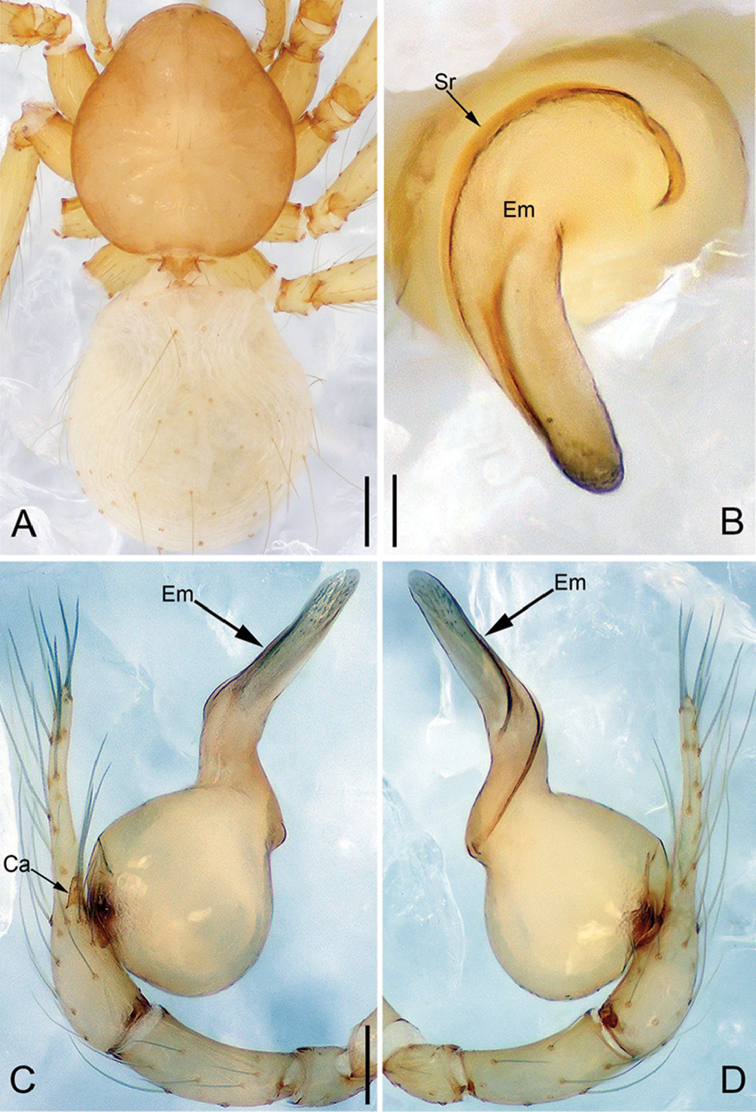
*Pinelemahuoyan* Zhao & Li, sp. n., male holotype. **A** Habitus, dorsal view **B** Embolus, apical view **C** Palp, prolateral view **D** Palp, retrolateral view. Scale bars: 0.2 mm (**A**), 0.05 mm (**B**), 0.1 mm (**C–D**).

*Pinelemahuoyan* Zhao & Li, sp. n. differs from all other congeners of *P.bailongensis* species group by following combination of characters: eyeless (Figure 7A), embolus with small distal-retrolateral apophyses (arrow in Figure 6D), and Esl/El ratio 0.72–0.78 (n = 5, mean: 0.76). In other congeners, combination of relative characters are either eyeless and no apophyses, or eyes distinct and no apophyses, or eyes distinct and apophyses present, and Esl/El ratio 0.33–0.67.

##### Description.

**Male (holotype)**: Total length 1.58. Carapace 0.75 long, 0.64 wide. Abdomen 0.84 long, 0.71 wide. Carapace brown (Figure 7A). Eyeless (Figure 7A). Chelicerae, legs, labium, and endites brown. Sternum bright brown with sparse setae. Leg measurements: I 6.23 (1.84, 0.28, 1.96, 1.39, 0.76); II 5.42 (1.64, 0.27, 1.70, 1.18, 0.63); III 3.53 (1.28, 0.22, 1.18, 0.85, 0.53); IV 4.98 (1.60, 0.22, 1.44, 1.10, 0.62). Abdomen pale yellow with a few long setae (Figure 7A).

Palp: Tibia 2.0 times longer than patella, cymbium 2.2 times longer than tibia, cymbial apophysis dark brown and cone-shaped (Figure 7C); bulb shaped as in Figure 7D; embolus with numerous small apophyses distally (arrow in Figure 6D), spiral ridge brown (Figure 7B), El/Bl ratio 1.42, and Esl/El ratio 0.76.

**Figure 8.**
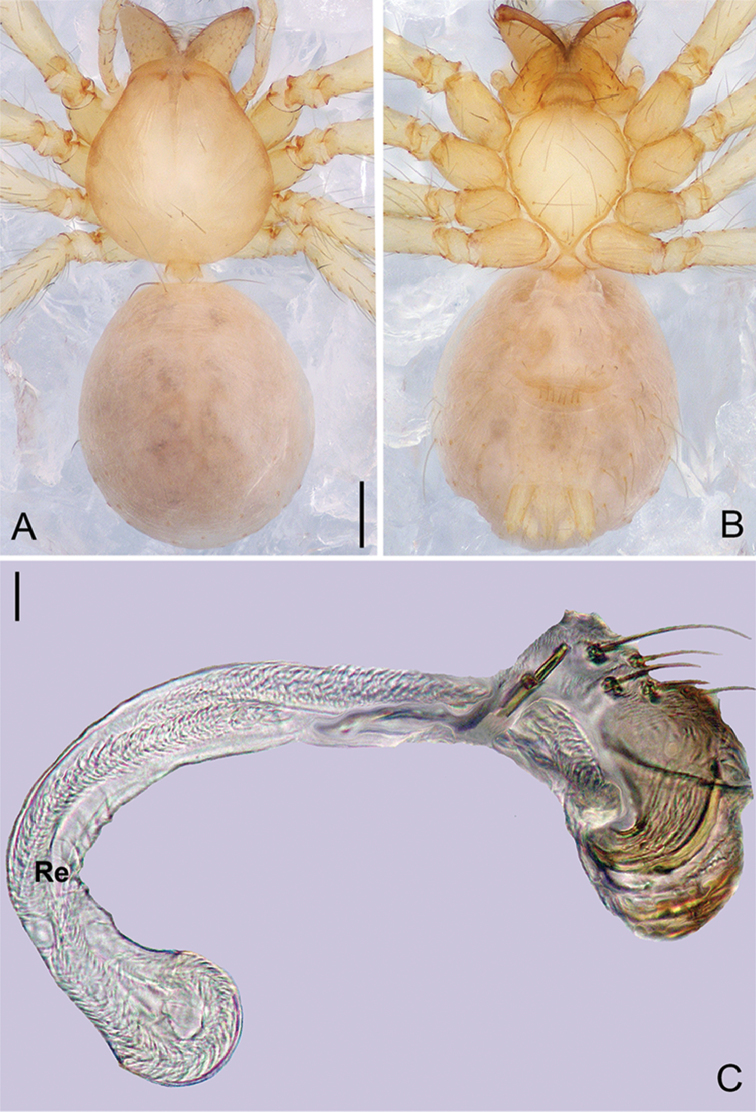
*Pinelemahuoyan* Zhao & Li, sp. n., female paratype. **A** Habitus, dorsal view **B** Habitus, ventral view **C** Endogyne, lateral view. Scale bars: 0.2 mm (**A–B**), 0.05 mm (**C**).

**Female**: Total length 1.48. Carapace 0.65 long, 0.58 wide. Abdomen 0.85 long, 0.71 wide. Coloration as in male (Figure 8A, B). Leg measurements: I 5.89 (1.76, 0.25, 1.88, 1.25, 0.75); II 5.26 (1.62, 0.25, 1.64, 1.08, 0.67); III 3.75 (1.19, 0.23, 1.13, 0.73, 0.47); IV 4.64 (1.50, 0.24, 1.34, 0.96, 0.60). Abdomen light brown. Insemination duct with a membranous tube (Figure 8C); receptacle as wide as insemination duct, U-shaped, slightly swollen at end (Figure 8C).

##### Variation.

In 4♂ paratypes: El/Bl ratio 1.38–1.43, Esl/El ratio 0.72–0.78.

##### Distribution.

China (Guangxi, Figure 31), known only from the type locality.

#### Pinelema liangxi

Taxon classificationAnimaliaAraneaeTelemidae

(Zhu & Chen, 2002) comb. n.

**Figure 9.**
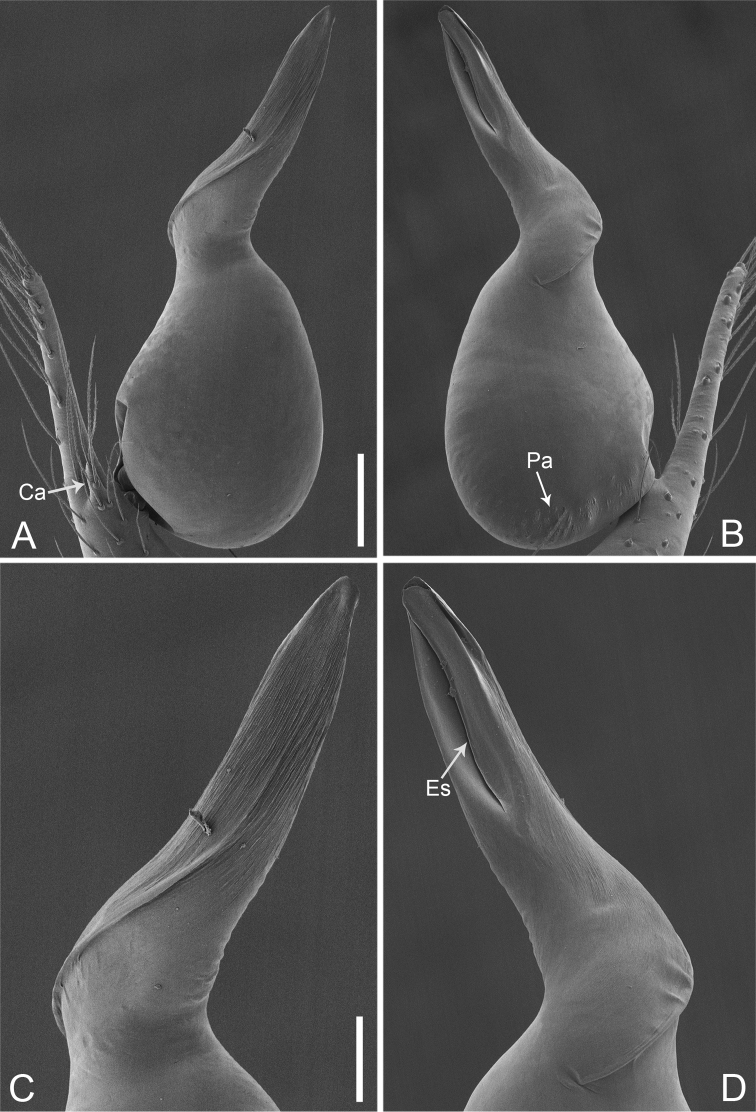
*Pinelemaliangxi*, male. **A** Palp, prolateral view **B** Palp, retrolateral view **C** Embolus, prolateral view **D** Embolus, retrolateral view. Scale bars: 0.1 mm (**A–B**), 0.05 mm (**C–D**).

**Figure 10.**
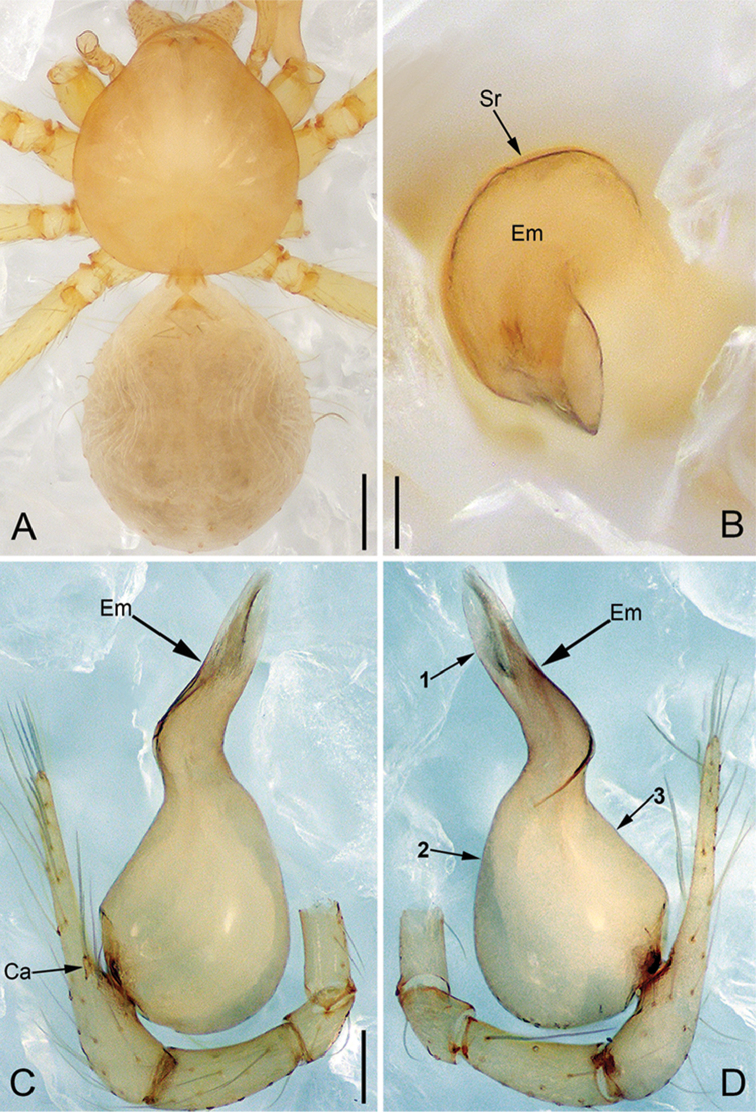
*Pinelemaliangxi*, male. **A** Habitus, dorsal view **B** Embolus, apical view **C** Palp, prolateral view **D** Palp, retrolateral view. Scale bars: 0.2 mm (**A**), 0.05 mm (**B**), 0.1 mm (**C–D**).

**Figure 11.**
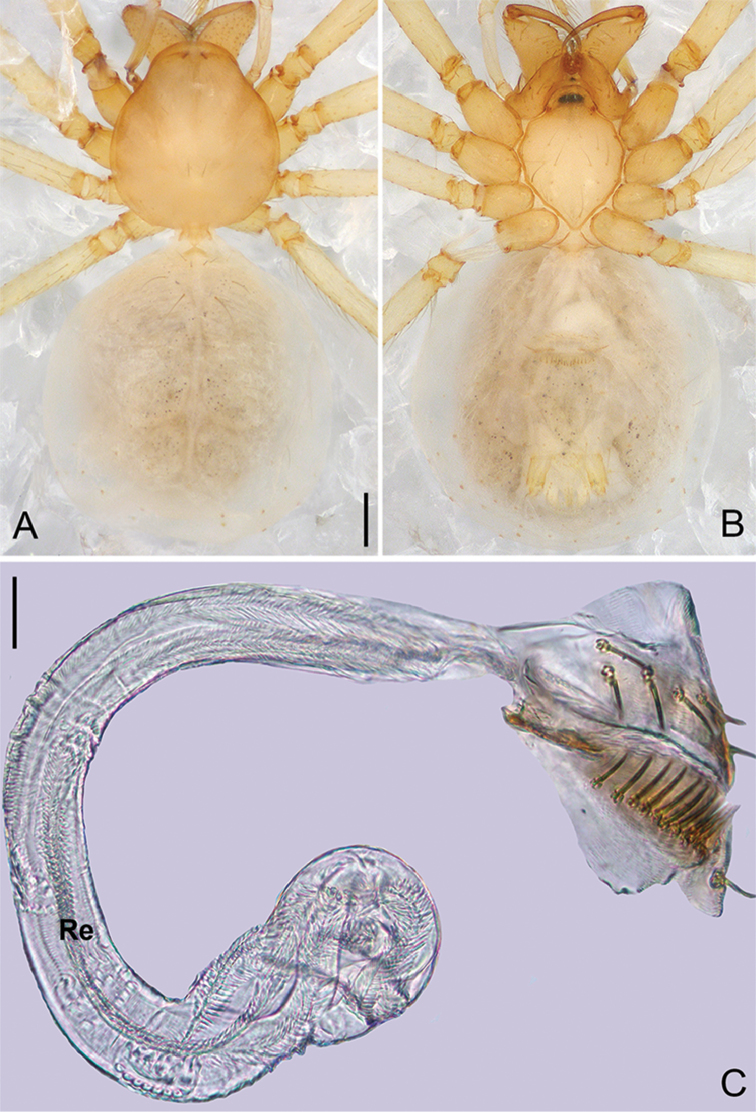
*Pinelemaliangxi*, female. **A** Habitus, dorsal view **B** Habitus, ventral view **C** Endogyne, lateral view. Scale bars: 0.2 mm (**A–B**), 0.05 mm (**C**).

Figs 9 , 10 , 11 , 31

Telema liangxi : Zhu and Chen 2002: 82, figs 1–7 (♂♀); Chen and Zhu 2009: 1709, figure 3E, M–N (♂♀).

##### Type material.

holotype ♂ (MHBU), China, Guizhou Province, Qiannan Prefecture, Libo County, Maolan National Nature Reserve: Liangxi Cave, 24.5°N, 100.2°E, 5.XI.1999, H. Chen leg. Paratypes (MHBU): 2♀, same data as holotype. Not examined.

##### Material examined.

5♂ and 8♀ (including five molecular vouchers, IZCAS) from the type locality, 25°12'N, 108°00'E, 15.III.2011, C. Wang and L. Lin leg.

##### Diagnosis.

*Pinelemaliangxi* resembles *P.strentarsi* (see Figs 15–17 and Lin and Li 2010: 23, figs 14–15) but can be distinguished by following characters: embolus curved (arrow 1 in Figure 10D) (embolus straight in *P.strentarsi*), bulb not protruding ventro-subdistally (arrow 2 in Figure 10D) (bulb protruding ventro-subdistally in *P.strentarsi*), and bulb not curved dorso-medially (arrow 3 in Figure 10D) (bulb curved dorso-medially in *P.strentarsi*).

##### Description.

Male palp: Cymbial apophysis light brown (Figure 10C); bulb with a few papillae proximo-retrolaterally (Figure 9B); El/Bl ratio 1.14–1.25 (n = 5, mean: 1.20, Suppl. material 1: Figure S5), Esl/El ratio 0.49–0.51 (n = 5, mean: 0.50, Suppl. material 1: Figure S5). Endogyne: receptacle thin and long, U-shaped (see Figure 11C and Chen and Zhu 2009: figure 3M, N). For more detailed descriptions, see Zhu and Chen (2002).

##### Comments.

Because this species shares similar morphological characters with *P.bailongensis*, such as the long, tube-shaped embolus (see Figs 9A–D, 10C–D and Zhu and Chen 2002: figs 5–6), the presence of a distinct cymbial apophysis in the male palp prolaterally (see Figs 9A, 10C and Zhu and Chen 2002: figure 5), and the U-shaped and medially strongly curved receptacle (Figure 11C), it is transferred to *Pinelema*.

##### Remarks.

The coordinates of the type locality of this species in Zhu and Chen (2002) is wrong because they refer to the place located approximately 700 kilometers from Liangxi Cave. The coordinate information reported here is confirmed by Dr H. Chen who collected type material.

##### Distribution.

China (Guizhou, Figure 31), known only from the type locality.

#### Pinelema lizhuang

Taxon classificationAnimaliaAraneaeTelemidae

Zhao & Li sp. n.

http://zoobank.org/8C9BA784-6DBB-4DA7-B7A8-FAB0F9015D28

**Figure 12.**
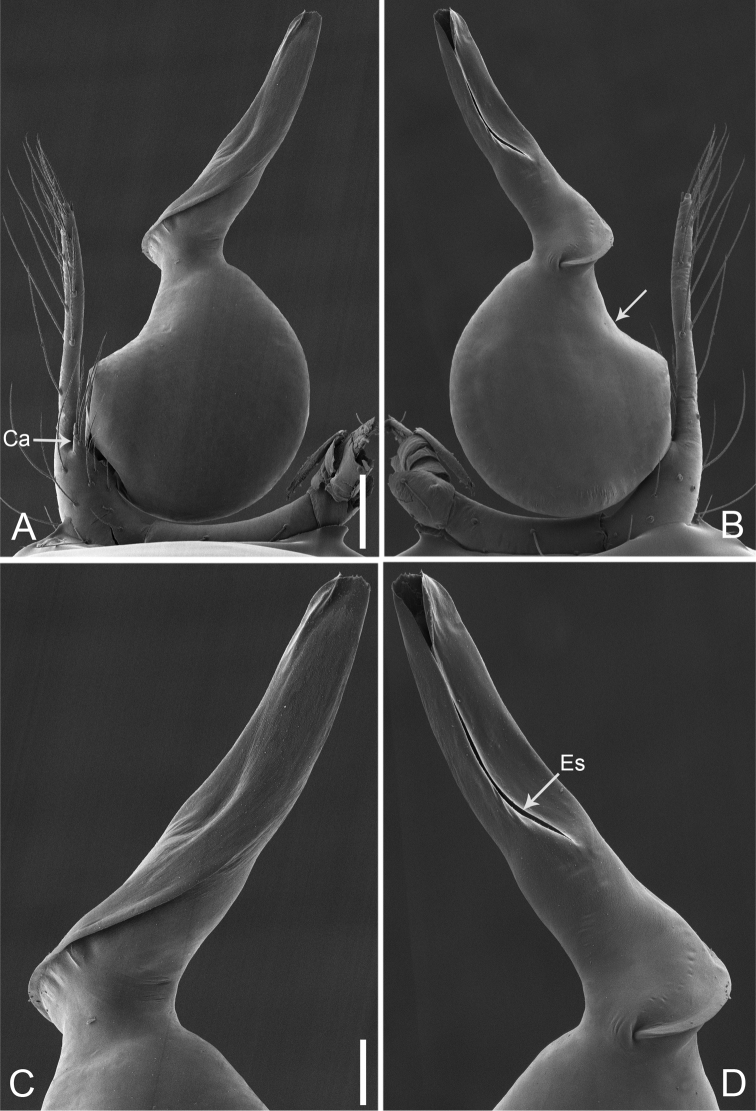
*Pinelemalizhuang* Zhao & Li, sp. n., male. **A** Palp, prolateral view **B** Palp, retrolateral view **C** Embolus, prolateral view **D** Embolus, retrolateral view. Scale bars: 0.1 mm (**A–B**), 0.05 mm (**C–D**).

**Figure 13.**
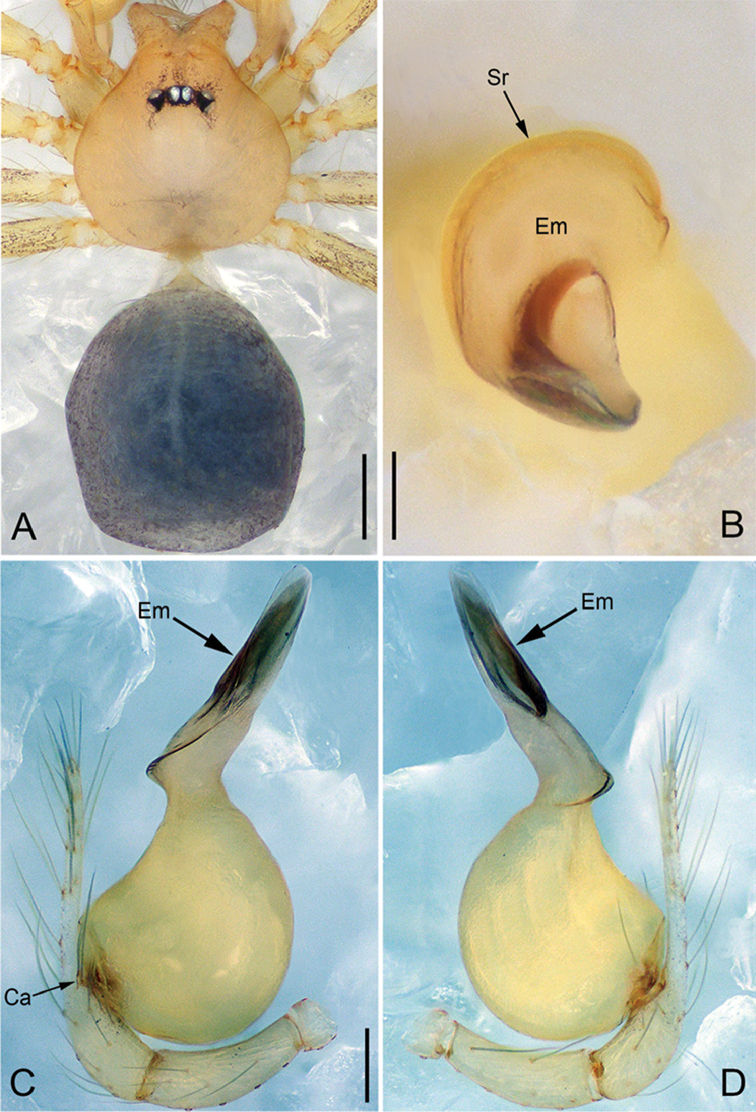
*Pinelemalizhuang* Zhao & Li, sp. n., male holotype. **A** Habitus, dorsal view **B** Embolus, apical view **C** Palp, prolateral view **D** Palp, retrolateral view. Scale bars: 0.2 mm (**A**), 0.05 mm (**B**), 0.1 mm (**C–D**).

**Figure 14.**
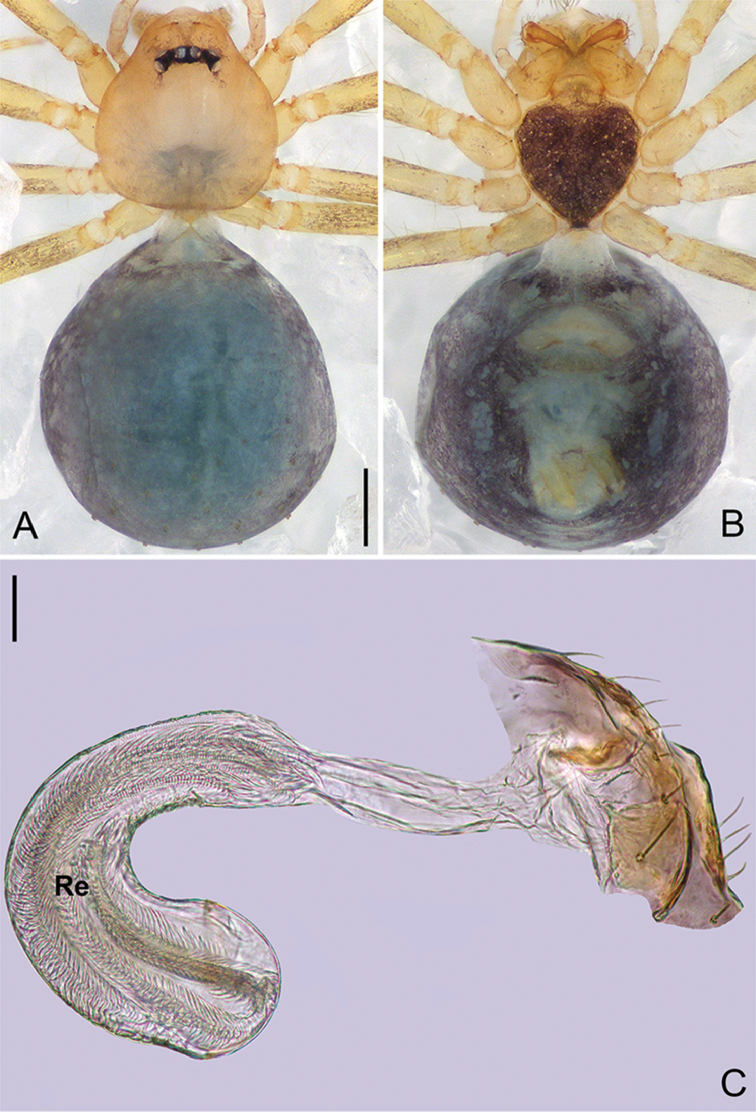
*Pinelemalizhuang* Zhao & Li, sp. n., female paratype. **A** Habitus, dorsal view **B** Habitus, ventral view **C** Endogyne, lateral view. Scale bars: 0.2 mm (**A–B**), 0.05 mm (**C**).

Figs 12 , 13 , 14 , 31

##### Type material.

**Holotype** ♂ (IZCAS): China, Guangxi Zhuang Autonomous Region, Hechi Prefecture, Nandan County, Liuzai Town, Lizhuang Village, Cave without a name, 25°18.490﻿'N, 107°22.910'E, 828 m, 28.I.2015, Y. Li and Z. Chen leg. **Paratypes** (IZCAS): 5♂ and 5♀, same data as holotype.

##### Other material examined.

4♀ (molecular vouchers, IZCAS), same data as holotype.

##### Etymology.

The species epithet refers to the type locality; noun.

##### Diagnosis.

This species resembles *P.wenyang* Zhao & Li, sp. n. (Figs 21–23) but can be distinguished by following characters: bulb curved dorso-medially (Figure 13D) (bulb curved dorso-subdistally in *P.wenyang* Zhao & Li, sp. n.), larger Esl/El ratio (0.63–0.67, n = 6, mean = 0.65, Suppl. material 1: Figure S6) (smaller Esl/El ratio 0.51–0.55, n = 5, mean = 0.53 in *P.wenyang* Zhao & Li, sp. n.), and smaller El/Bl ratio (1.24–1.31, n = 6, mean: 1.27, Suppl. material 1: Figure S6) (larger El/Bl ratio 1.47–1.54, n = 5, mean: 1.49 in *P.wenyang* Zhao & Li, sp. n.).

##### Description.

**Male (holotype)**: Total length 1.20. Carapace 0.50 long, 0.51 wide. Abdomen 0.67 long, 0.56 wide. Carapace pale brown (Figure 13A). Six eyes ringed with black (Figure 13A), clypeus height 0.11, ocular quadrangle 0.15 wide. Chelicerae, legs, labium, and endites light yellow with dark brown dots. Sternum dark brown with sparse setae. Leg measurements: I 3.83 (1.13, 0.19, 1.18, 0.78, 0.55); II 3.15 (0.92, 0.19, 0.96, 0.59, 0.49); III 2.29 (0.67, 0.16, 0.63, 0.43, 0.40); IV 2.36 (0.87, 0.18, 0.78, 0.53, 0.42). Abdomen purple-bluish (Figure 13A).

Palp: Tibia 2.9 times longer than patella, cymbium 2.0 times longer than tibia, cymbial apophysis cone-shaped (Figure 13C); bulb hemispherical (Figure 13C, D); spiral ridge brown (Figure 13B), El/Bl ratio 1.26 (Figure 13D), the distal part of embolus black (Figure 13C, D), and Esl/El ratio 0.65 (Figure 13D).

**Female**: Total length 1.39. Carapace 0.50 long, 0.48 wide. Abdomen 0.85 long, 0.73 wide. Coloration as in male (Figure 14A–B). Six eyes, well-developed, clypeus height 0.11, ocular quadrangle 0.17 wide. Leg measurements: I 3.42 (1.00, 0.19, 1.05, 0.65, 0.53); II 2.86 (0.85, 0.19, 0.85, 0.53, 0.44); III 2.06 (0.63, 0.19, 0.53, 0.37, 0.34); IV 2.68 (0.85, 0.19, 0.76, 0.48, 0.40). Abdomen purple-bluish (Figure 14A). Insemination duct as wide as receptacle (Figure 14C); receptacle C-shaped with several membranous tubes inside (Figure 14C).

##### Variation.

In 5♂ paratypes: El/Bl ratio 1.24–1.31, Esl/El ratio 0.63–0.67.

##### Distribution.

China (Guangxi, Figure 31), known only from the type locality.

#### Pinelema strentarsi

Taxon classificationAnimaliaAraneaeTelemidae

(Lin & Li, 2010) comb. n.

**Figure 15.**
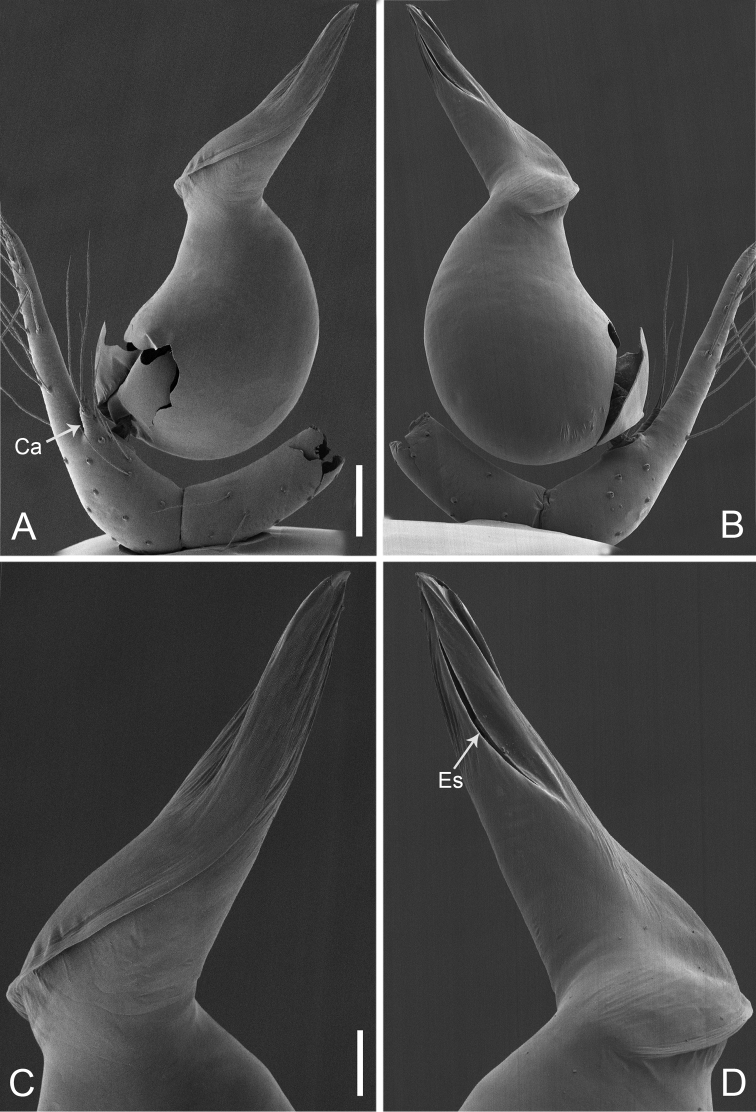
*Pinelemastrentarsi*, male. **A** Palp, prolateral view **B** Palp, retrolateral view **C** Embolus, prolateral view **D** Embolus, retrolateral view. Scale bars: 0.1 mm (**A–B**), 0.05 mm (**C–D**).

**Figure 16.**
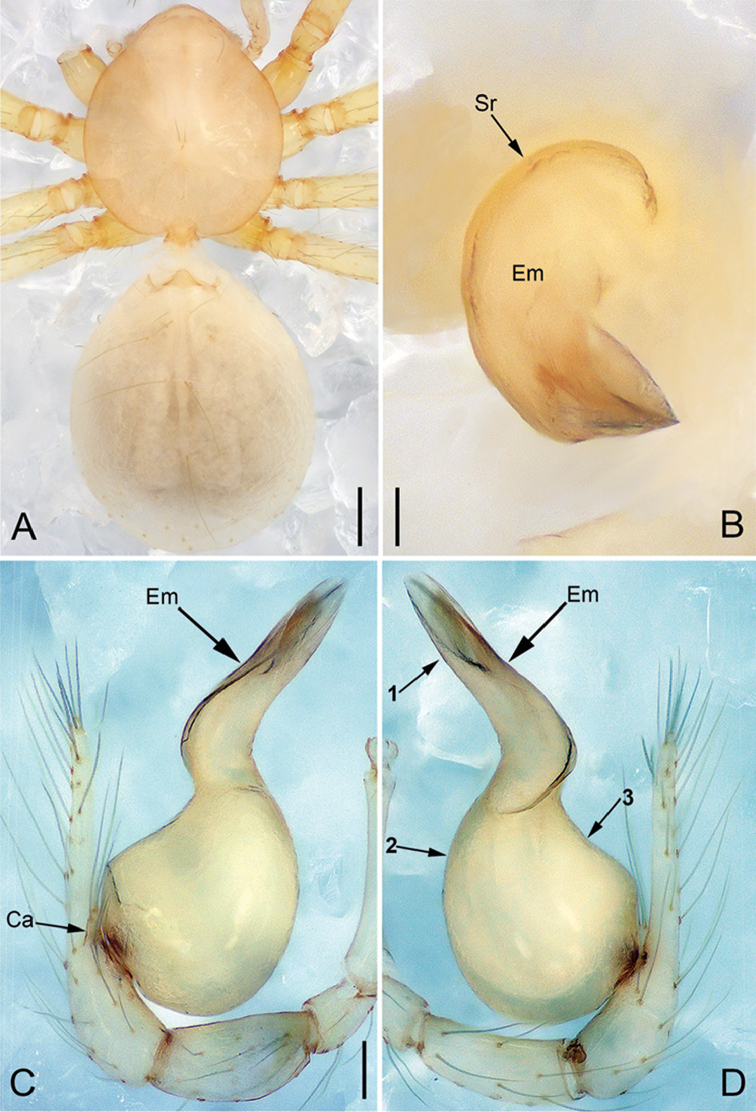
*Pinelemastrentarsi*, male. **A** Habitus, dorsal view **B** Embolus, apical view **C** Palp, prolateral view **D** Palp, retrolateral view. Scale bars: 0.2 mm (**A**), 0.05 mm (**B**), 0.1 mm (**C–D**).

**Figure 17.**
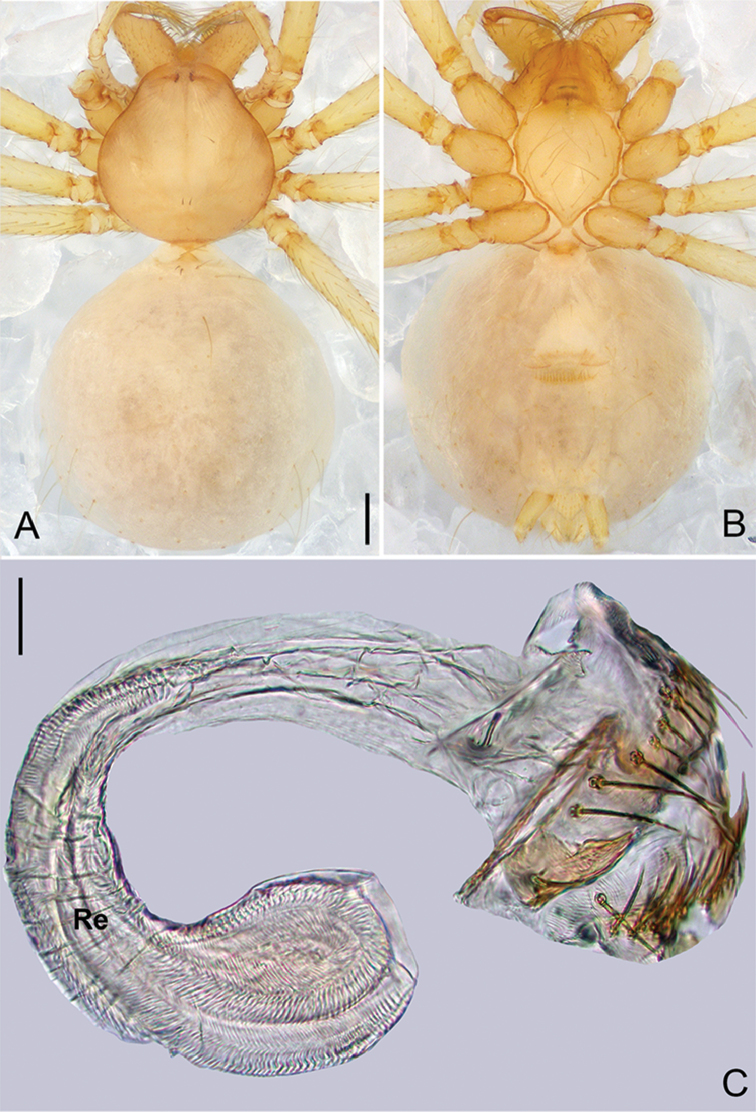
*Pinelemastrentarsi*, female. **A** Habitus, dorsal view **B** Habitus, ventral view **C** Endogyne, lateral view. Scale bars: 0.2 mm (**A–B**), 0.05 mm (**C**).

Figs 15 , 16 , 17 , 31

Telema strentarsi : Lin and Li 2010: 23, figs 14–15 (♂♀).

##### Type material.

**examined.** Holotype ♂ (IZCAS): China, Guangxi Zhuang Autonomous Region, Hechi Prefecture, Dahua County, Jiangnan Township, Huangniu Cave, 23°55.120'N, 107°37.479'E, 8.III.2007, J. Liu and Y. Lin leg. Paratypes: 1♂ and 6♀ (IZCAS), same data as holotype.

##### Other material examined.

4♂ and 5♀ (molecular vouchers, IZCAS) from the type locality, 175 m, 10.IV.2017, Z. Chen leg.

##### Diagnosis.

This species resembles *P.liangxi* (see Figs 9–11, Zhu and Chen 2002: 82, figs 1–7 and Chen and Zhu 2009: 1709, figure 3E, M–N) but can be differentiated by following characters: embolus straight (arrow 1 in Figure 16D) (embolus curved in *P.liangxi*), bulb protruding ventro-subdistally (arrow 2 in Figure 16D) (bulb not protruding ventro-subdistally in *P.liangxi*), and bulb slightly curved dorso-medially (arrow 3 in Figure 16D) (bulb not curved dorso-medially in *P.liangxi*).

##### Description.

Male palp: Cymbial apophysis finger-like (Figs 15A, 16C); spiral ridge pale brown (Figure 16B), El/Bl ratio 1.15–1.21 (n = 4, mean: 1.18, Suppl. material 1: Figure S7), Esl/El ratio 0.48–0.49 (n = 4, mean: 0.48, Suppl. material 1: Figure S7), Female endogyne: insemination duct wider than receptacle, receptacle comma-shaped (Figure 17C). For more detailed descriptions, see Lin and Li (2010).

##### Comments.

This species is transferred to *Pinelema*, because it shares similar morphological characters with *P.bailongensis*, such as the long, tube-like embolus (see Figs 15A–D, 16C–D and Lin and Li 2010: figure 14D–F), the presence of a distinct cymbial apophysis in male palp prolaterally (see Figs 15A, 16C and Lin and Li 2010: figure 14E), and the U-shaped and medially strongly curved receptacle (see Figure 17C and Lin and Li 2010: figure 15F).

##### Distribution.

China (Guangxi, Figure 31), known only from the type locality.

#### Pinelema wangshang

Taxon classificationAnimaliaAraneaeTelemidae

Zhao & Li sp. n.

http://zoobank.org/4EE41668-F748-4EC3-AA54-FAF109BB407B

**Figure 18.**
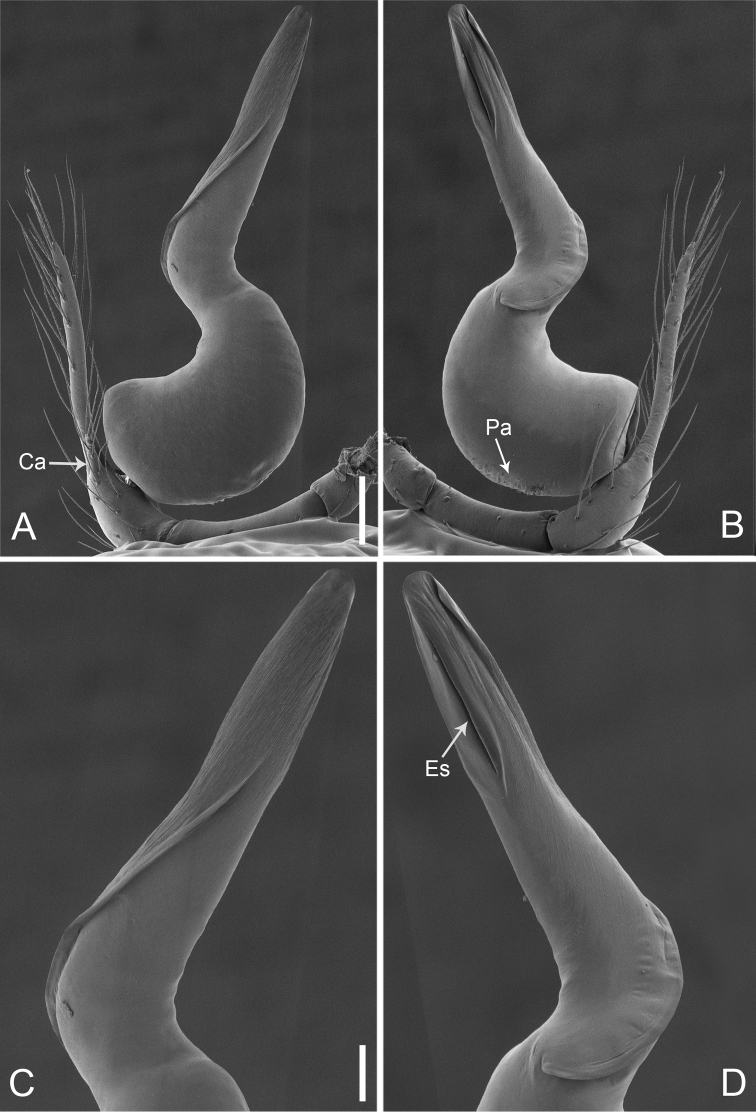
*Pinelemawangshang* Zhao & Li, sp. n., male. **A** Palp, prolateral view **B** Palp, retrolateral view **C** Embolus, prolateral view **D** Embolus, retrolateral view. Scale bars: 0.1 mm (**A–B**), 0.05 mm (**C–D**).

**Figure 19.**
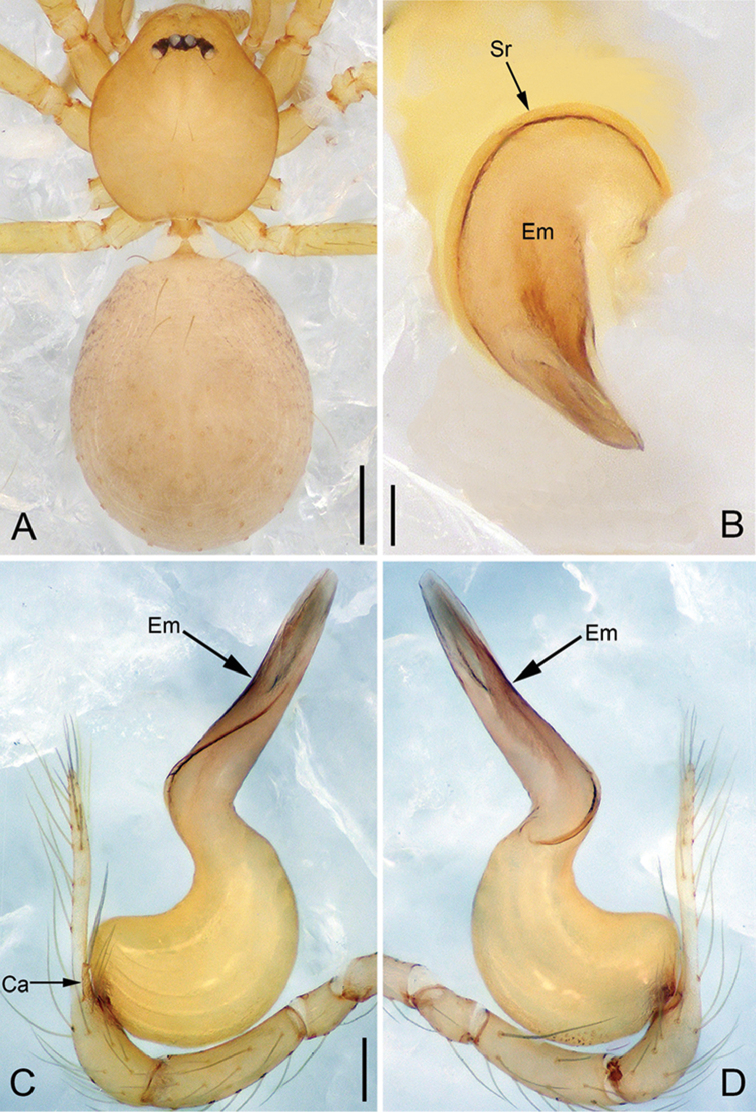
*Pinelemawangshang* Zhao & Li, sp. n., male holotype. **A** Habitus, dorsal view **B** Embolus, apical view **C** Palp, prolateral view **D** Palp, retrolateral view. Scale bars: 0.2 mm (**A**), 0.05 mm (**B**), 0.1 mm (**C–D**).

**Figure 20.**
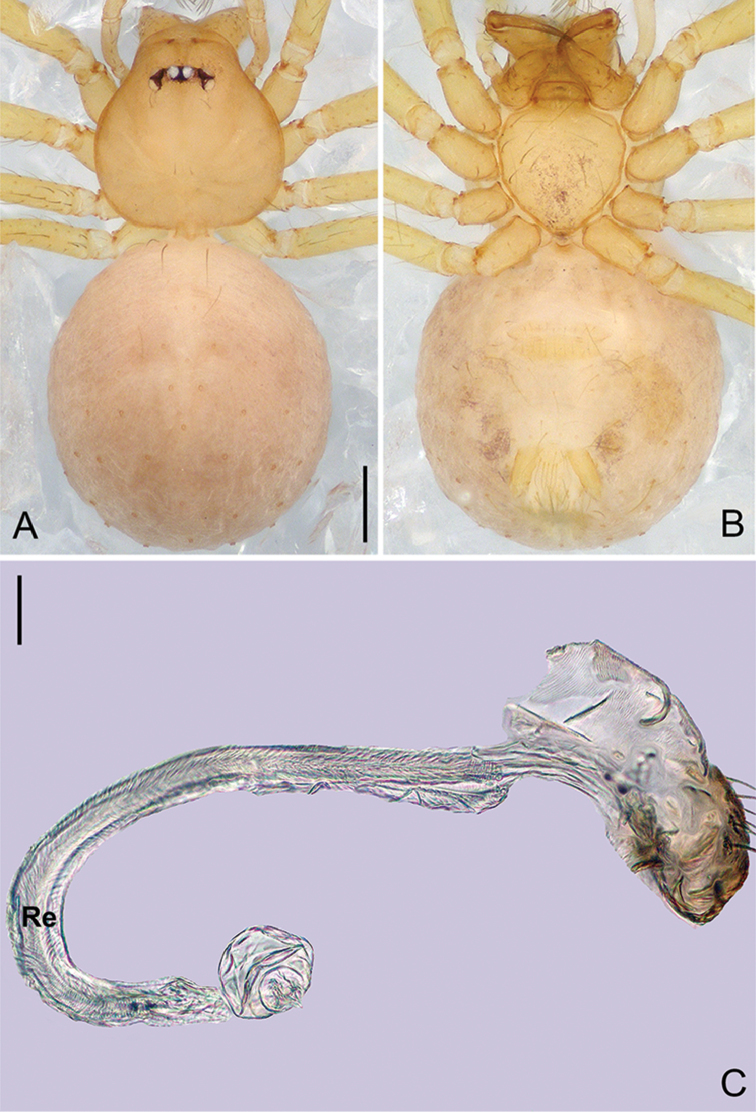
*Pinelemawangshang* Zhao & Li, sp. n., female paratype. **A** Habitus, dorsal view **B** Habitus, ventral view **C** Endogyne, lateral view. Scale bars: 0.2 mm (**A–B**), 0.05 mm (**C**).

Figs 18 , 19 , 20 , 31

##### Type material.

**Holotype** ♂ (IZCAS): China, Guangxi Zhuang Autonomous Region, Hechi Prefecture, Nandan County, Lihu Town, Wangshang Village, Wangshang Cave, 25°05.300'N, 107°38.420'E, 602 m, 1.II.2015, Y. Li and Z. Chen leg. **Paratypes** (IZCAS): 4♂ and 5♀, same data as holotype.

##### Other material examined.

4♀ (molecular vouchers, IZCAS), same data as holotype.

##### Etymology.

The species epithet refers to the type locality; noun.

##### Diagnosis.

This species resembles *P.yunchuni* Zhao & Li, sp. n. (Figs 25–27) but can be distinguished by following characters: bulb kidney-shaped (Figs 18A, B, 19C, D) (bulb pear-shaped in *P.yunchuni* Zhao & Li, sp. n.), curved dorso-medially (Figs 18A, B, 19C, D) (bulb not curved dorso-medially in *P.yunchuni* Zhao & Li, sp. n.), smaller El/Bl ratio (1.37–1.45, n = 5, mean: 1.41, Suppl. material 1: Figure S8) (larger El/Bl ratio 1.78–1.82, n = 4, mean: 1.80 in *P.yunchuni* Zhao & Li, sp. n.), and receptacle J-shaped (Figure 20C) (receptacle U-shaped in *P.yunchuni* Zhao & Li, sp. n.).

##### Description.

**Male (holotype)**: Total length 1.39. Carapace 0.54 long, 0.48 wide. Abdomen 0.79 long, 0.60 wide. Carapace brown (Figure 19A). Six eyes ringed with black, clypeus height 0.08, ocular quadrangle 0.15 wide (Figure 19A). Chelicerae, legs, labium, and endites yellow. Sternum light brown. Leg measurements: I 4.41 (1.25, 0.19, 1.41, 0.94, 0.62); II 3.78 (1.11, 0.19, 1.20, 0.75, 0.53); III 2.72 (0.85, 0.18, 0.76, 0.50, 0.43); IV 3.29 (1.05, 0.19, 0.96, 0.63, 0.46). Abdomen oval and light brown (Figure 19A).

Palp: Tibia 3 times longer than patella, cymbium 2 times longer than tibia, cymbial apophysis long and brown (Figure 19C); bulb with a U-shaped curve dorso-medially (Figs 18A–B, 19C–D) and with papillae proximo-retrolaterally (Figure 18B); embolus long and tube-like, spiral ridge distinct and brown (Figure 19B), El/Bl ratio 1.37 (Figure 19D), and Esl/El ratio 0.43 (Figure 19D).

**Female**: Total length 1.38. Carapace 0.54 long, 0.51 wide. Abdomen 0.84 long, 0.76 wide. Coloration as in male (Figure 20A–B). Six eyes ringed with black, clypeus height 0.11, ocular quadrangle 0.17 wide (Figure 20A). Leg measurements: I 4.22 (1.27, 0.18, 1.33, 0.85, 0.59); II 3.68 (1.20, 0.19, 1.08, 0.71, 0.50); III 2.69 (0.85, 0.18, 0.75, 0.50, 0.41); IV 3.37 (1.03, 0.19, 1.00, 0.67, 0.48). Insemination duct short (Figure 20C); receptacle J-shaped, as narrow as insemination duct, end globular (Figure 20C).

##### Variation.

In 4♂ paratypes: El/Bl ratio 1.37–1.45, Esl/El ratio 0.42–0.43.

##### Distribution.

China (Guangxi, Figure 31), known only from the type locality.

#### Pinelema wenyang

Taxon classificationAnimaliaAraneaeTelemidae

Zhao & Li sp. n.

http://zoobank.org/81E4223F-A71B-4A14-A615-A7C26306C085

**Figure 21.**
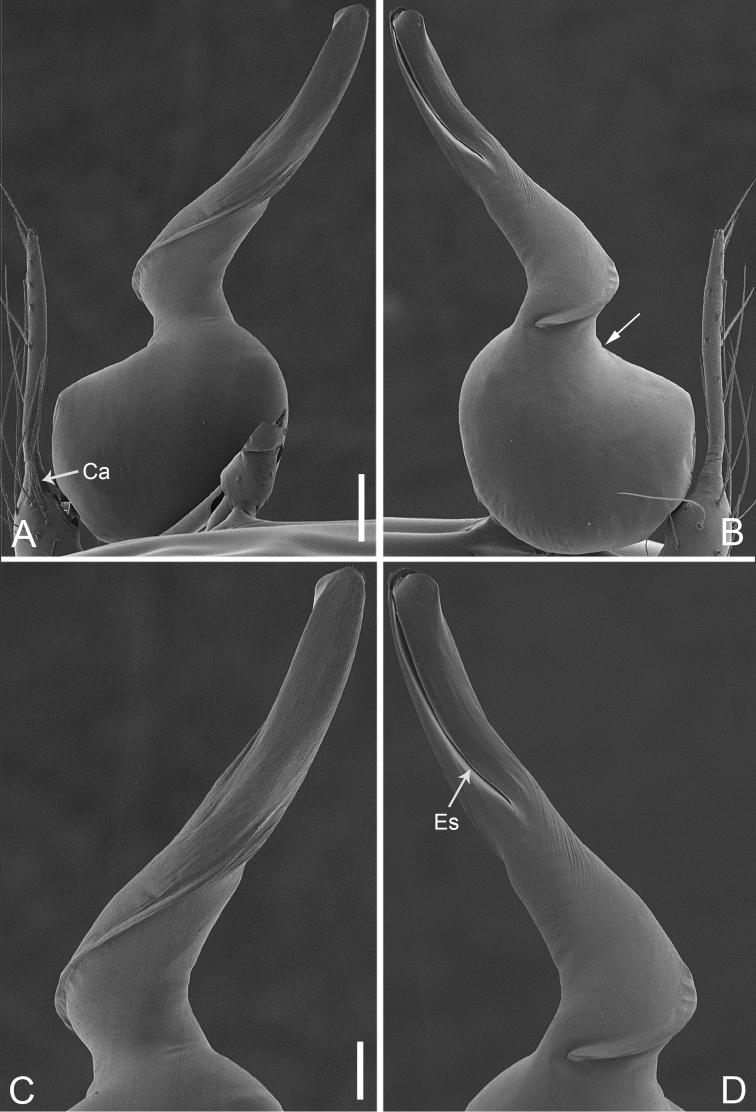
*Pinelemawenyang* Zhao & Li, sp. n., male. **A** Palp, prolateral view **B** Palp, retrolateral view **C** Embolus, prolateral view **D** Embolus, retrolateral view. Scale bars: 0.1 mm (**A–B**), 0.05 mm (**C–D**).

**Figure 22.**
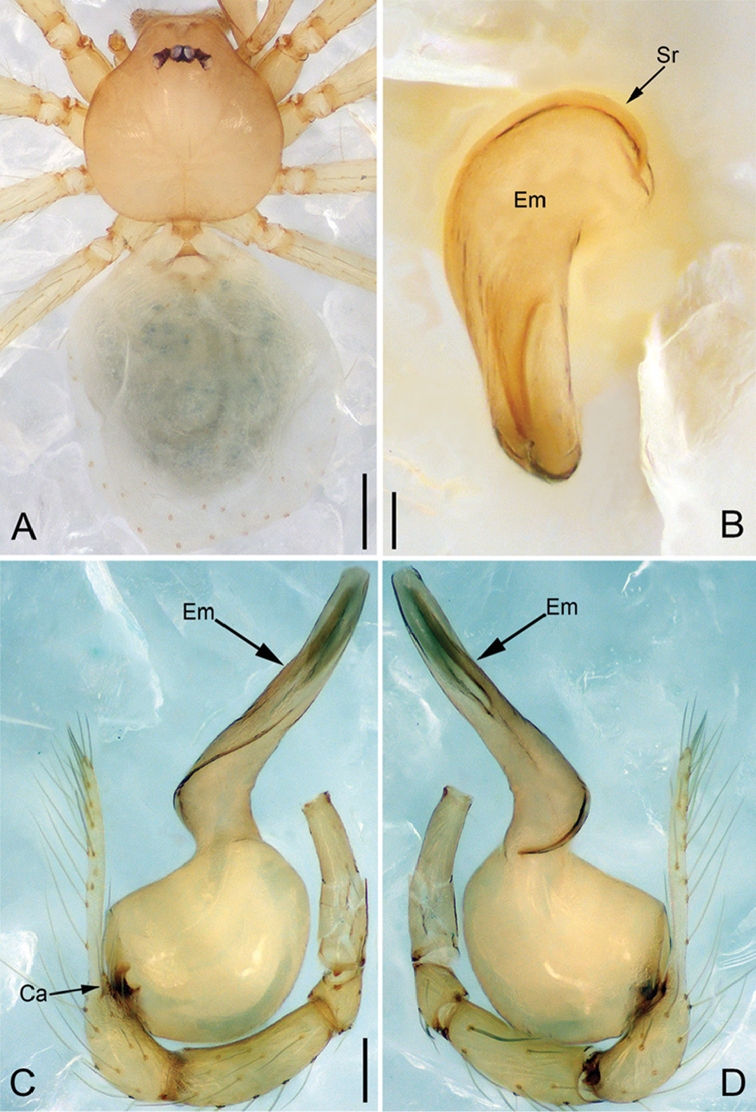
*Pinelemawenyang* Zhao & Li, sp. n., male holotype. **A** Habitus, dorsal view **B** Embolus, apical view **C** Palp, prolateral view **D** Palp, retrolateral view. Scale bars: 0.2 mm (**A**), 0.05 mm (**B**), 0.1 mm (**C–D**).

**Figure 23.**
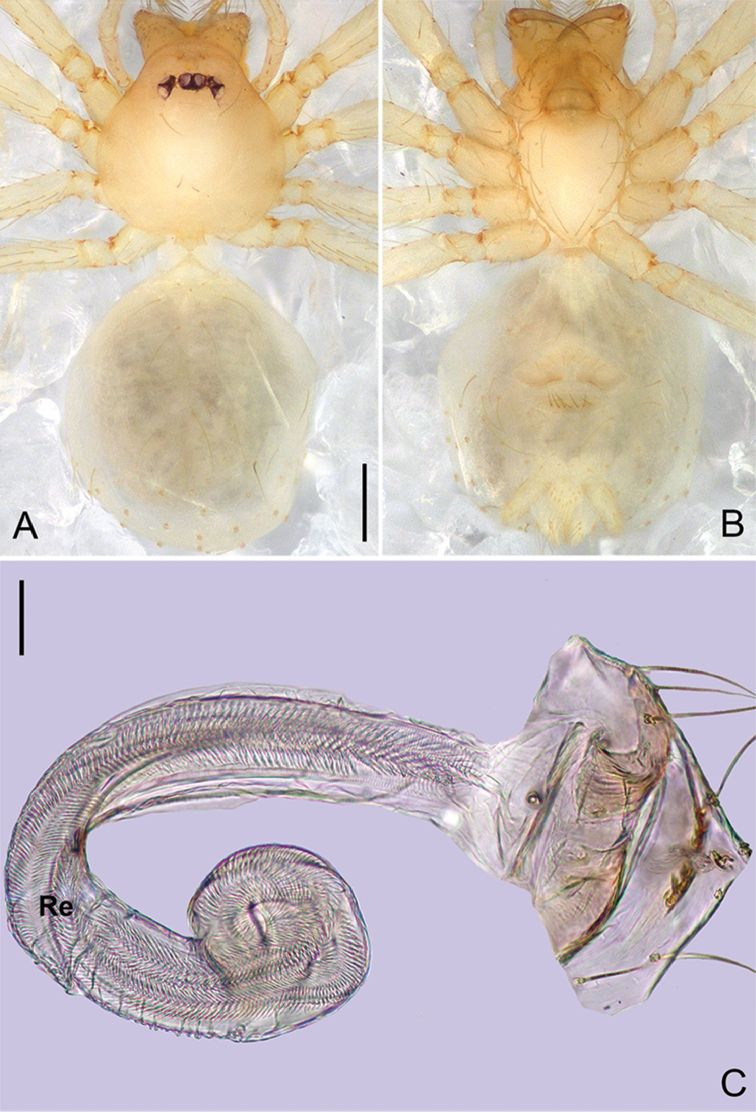
*Pinelemawenyang* Zhao & Li, sp. n., female paratype. **A** Habitus, dorsal view **B** Habitus, ventral view **C** Endogyne, lateral view. Scale bars: 0.2 mm (**A–B**), 0.05 mm (**C**).

Figs 21 , 22 , 23 , 31

##### Type material.

**Holotype** ♂ (IZCAS): China, Guangxi Zhuang Autonomous Region, Chongzuo Prefecture, Taiping Township: Wenyang Cave, 22°26.792'N, 107°24.134'E, 180 m, 13.IV.2017, Z. Chen leg. **Paratypes** (IZCAS): 4♂ and 5♀, same data as holotype.

##### Other material examined.

5♀ (molecular vouchers, IZCAS), same data as holotype.

##### Etymology.

The species epithet refers to the type locality; noun.

##### Diagnosis.

This species resembles *P.lizhuang* Zhao & Li, sp. n. (Figs 12–15) but can be distinguished by following characters: bulb curved dorso-distally (arrow in Figure 21B) (bulb curved dorso-medially in *P.lizhuang* Zhao & Li, sp. n.), smaller Esl/El ratio (0.51–0.55, n = 5, mean: 0.53, Suppl. material 1: Figure S9) (larger Esl/El ratio 0.63–0.67, n = 6, mean: 0.65 in *P.lizhuang* Zhao & Li, sp. n.), and larger El/Bl ratio (1.45–1.54, n = 5, mean: 1.49, Suppl. material 1: Figure S9) (smaller El/Bl ratio 1.23–1.29, n = 6, mean: 1.27 in *P.lizhuang* Zhao & Li, sp. n.).

##### Description.

**Male (holotype)**: Total length 1.33. Carapace 0.54 long, 0.53 wide. Abdomen 0.79 long, 0.68 wide. Carapace light brown (Figure 22A). Six eyes ringed with black, clypeus height 0.08, ocular quadrangle 0.16 wide (Figure 22A). Chelicerae, legs, labium, and endites light yellow. Sternum light brown. Leg measurements: I 4.55 (1.31, 0.22, 1.45, 0.94, 0.63); II 3.86 (1.13, 0.22, 1.19, 0.75, 0.57); III 2.93 (0.85, 0.20, 0.90, 0.53, 0.45); IV 3.34 (1.06, 0.22, 0.95, 0.63, 0.48). Abdomen grey with sparse, long setae (Figure 22A).

Palp: Tibia 3.2 times longer than patella, cymbium 2.1 times longer than tibia, cymbial apophysis approximately cone-shaped (Figs 21A, 22C); bulb strongly protruding ventro-subdistally (Figure 22D) and curved dorso-distally (arrow in Figure 22D); spiral ridge brown (Figure 22B), El/Bl ratio 1.54 (Figure 22D), and Esl/El ratio 0.52 (Figure 22D).

**Female**: Total length 1.33. Carapace 1.53 long, 0.50 wide. Abdomen 0.80 long, 0.67 wide. Coloration as in male (Figure 23A–B). Six eyes, well developed, clypeus height 0.09, ocular quadrangle 0.17 wide (Figure 23A). Leg measurements: I 4.23 (1.25, 0.19, 1.33, 0.83, 0.63); II 3.60 (1.08, 0.19, 1.13, 0.67, 0.53); III 2.69 (0.86, 0.17, 0.75, 0.48, 0.43); IV 3.16 (1.03, 0.18, 0.91, 0.59, 0.45). Insemination duct as wide as receptacle (Figure 23C); receptacle comma-shaped with a globular end (Figure 23C).

##### Variation.

In 4♂ paratypes: El/Bl ratio 1.45–1.50, Esl/El ratio 0.51–0.55.

##### Distribution.

China (Guangxi, Figure 31), known only from the type locality.

#### Pinelema xiushuiensis

Taxon classificationAnimaliaAraneaeTelemidae

Wang & Li, 2016

Figs 24 , 31

Pinelema xiushuiensis : Wang and Li 2016: 556, figs 9–12 (♂♀).

##### Material examined.

Holotype ♂ (IZCAS): China, Guangxi Zhuang Autonomous Region, Baise Prefecture, Pingguo County, Xingning Village, Xiushui Cave, T: 24°C, RH: 90%, 23°34.048'N, 107°40.777'E, 285 m, 3.VIII.2009, C. Wang and Z. Yao leg. Paratypes: 1♂ and 4♀ (IZCAS), same data as holotype. 5♂ and 5♀ (molecular vouchers, IZCAS), same data as holotype.

##### Diagnosis.

**Figure 24.**
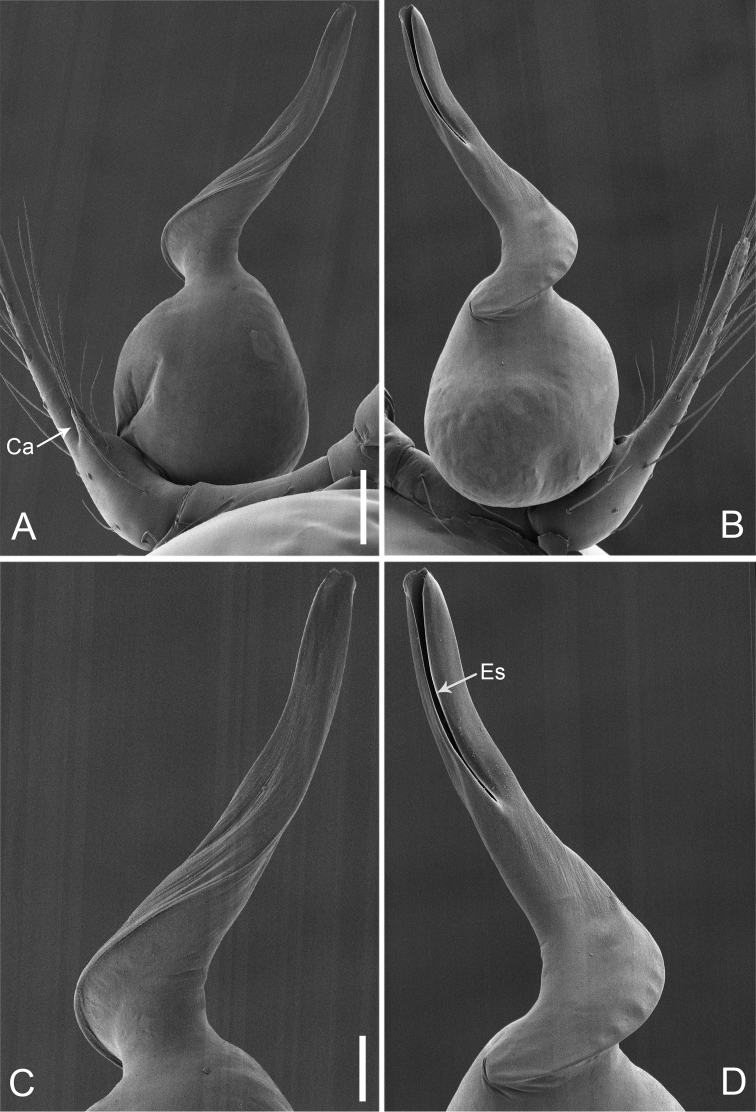
*Pinelemaxiushuiensis*, male. **A** Palp, prolateral view **B** Palp, retrolateral view **C** Embolus, prolateral view **D** Embolus, retrolateral view. Scale bars: 0.1 mm (**A–B**), 0.05 mm (**C–D**).

This species resembles *P.zhewang* (see Figs 28–30 and Chen and Zhu 2009: 1709, figure 3A–D, F–L, O–Q) but can be differentiated by following characters: cymbial apophysis long (Figure 24A) (cymbial apophysis very short in *P.zhewang*) and smaller Esl/El ratio (0.50–0.52, n = 5, mean: 0.51, Suppl. material 1: Figure S10) (larger Esl/El ratio 058–0.63, n = 4, mean: 0.60 in *P.zhewang*).

##### Description.

Male palp: Embolus bent (Figure 24A–D), El/Bl ratio 1.58–1.73 (n = 5, Suppl. material 1: Figure S10), and Esl/El ratio 0.50–0.52 (n = 5, Suppl. material 1: Figure S10). For more detailed descriptions, see Wang and Li (2016).

##### Distribution.

China (Guangxi, Figure 31), known only from the type locality.

#### Pinelema yunchuni

Taxon classificationAnimaliaAraneaeTelemidae

Zhao & Li sp. n.

http://zoobank.org/56E92945-7F34-47D1-9E91-2ED4C8B2EF46

**Figure 25.**
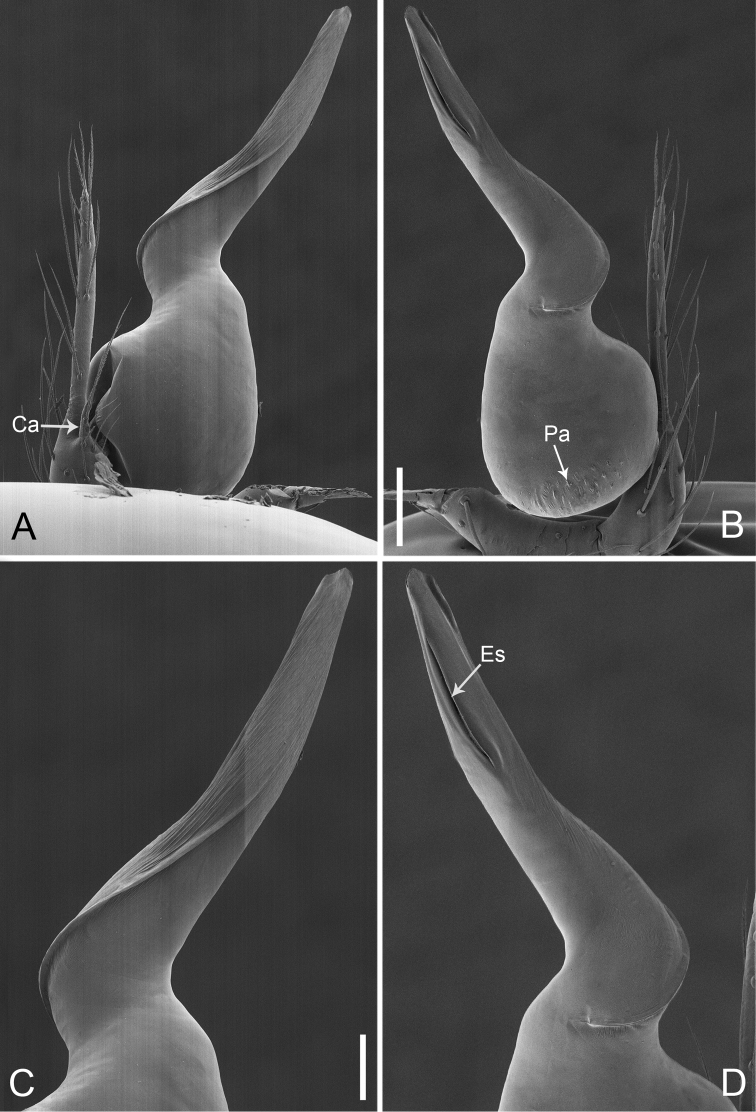
*Pinelemayunchuni* Zhao & Li, sp. n., male. **A** Palp, prolateral view **B** Palp, retrolateral view **C** Embolus, prolateral view **D** Embolus, retrolateral view. Scale bars: 0.1 mm (**A–B**), 0.05 mm (**C–D**).

**Figure 26.**
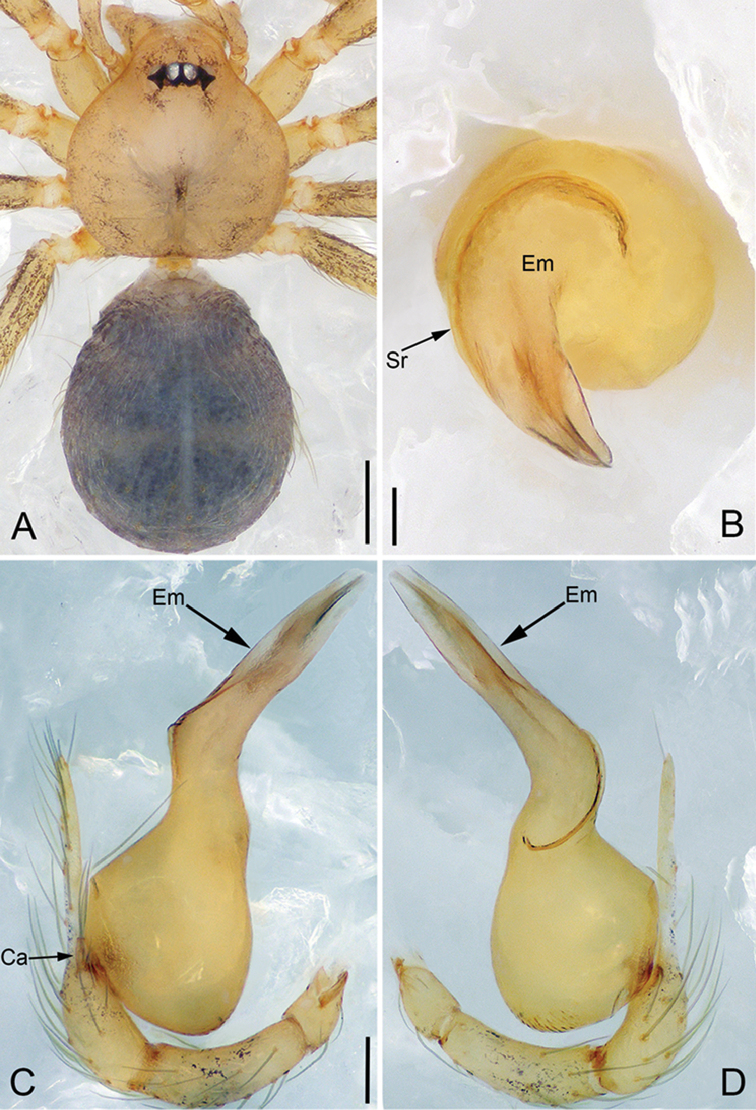
*Pinelemayunchuni* Zhao & Li, sp. n., male holotype. **A** Habitus, dorsal view **B** Embolus, apical view **C** Palp, prolateral view **D** Palp, retrolateral view. Scale bars: 0.2 mm (**A**), 0.05 mm (**B**), 0.1 mm (**C–D**).

**Figure 27.**
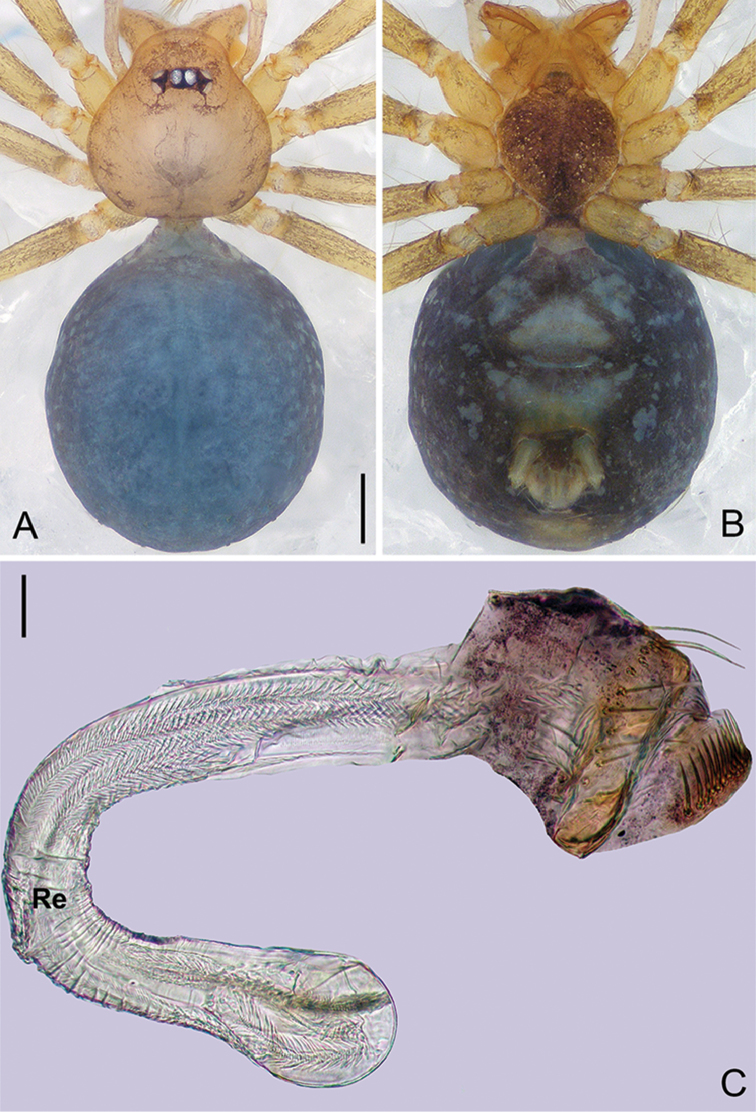
*Pinelemayunchuni* Zhao & Li, sp. n., female paratype. **A** Habitus, dorsal view **B** Habitus, ventral view **C** Endogyne, lateral view. Scale bars: 0.2 mm (**A–B**), 0.05 mm (**C**).

Figs 25 , 26 , 27 , 31

##### Type material.

**Holotype** ♂ (IZCAS): China, Guangxi Zhuang Autonomous Region, Hechi Prefecture, Du’an County, Gaoling Town, Sanlian Village, Cave without a name, 24°02.340'N, 108°03.720'E, 225 m, 12.III.2015, Y. Li and Z. Chen leg. **Paratypes** (IZCAS): 3♂ and 5♀, same data as holotype.

##### Other material examined.

5♀ (molecular vouchers, IZCAS), same data as holotype.

##### Etymology.

The specific name is a patronym in honor of the collector Yunchun Li.

##### Diagnosis.

This species resembles *P.wangshang* Zhao & Li, sp. n. (Figs 18–20) but can be distinguished by following characters: bulb pear-shaped (Figs 25A, B, 26C, D) (bulb kidney-shaped in *P.wangshang* Zhao & Li, sp. n.), not curved dorso-medially (Figure 26C, D) (bulb curved dorso-medially in *P.wangshang* Zhao & Li, sp. n.), larger El/Bl ratio (1.78–1.82, n = 4, mean: 1.80, Figs 25B, 26D) (smaller El/Bl ratio 1.37–1.45, n = 5, mean: 1.41 in *P.wangshang* Zhao & Li, sp. n.), and receptacle U-shaped (Figure 27C) (receptacle J-shaped in *P.wangshang* Zhao & Li, sp. n.).

##### Description.

**Male (holotype)**: Total length 1.27. Carapace 0.56 long, 0.53 wide. Abdomen 0.68 long, 0.56 wide. Carapace light brown with black speckles (Figure 26A). Six eyes ringed with black, clypeus height 0.10, ocular quadrangle 0.17 wide (Figure 26A). Chelicerae, legs, labium, and endites light brown. Sternum dark brown. Leg measurements: I 4.43 (1.33, 0.22, 1.41, 0.86, 0.61); II 3.63 (1.08, 0.21, 1.14, 0.67, 0.53); III 2.64 (0.81, 0.19, 0.76, 0.45, 0.43); IV 3.21 (1.04, 0.19, 0.94, 0.59, 0.45). Abdomen blue with sparse setae (Figure 26A).

Palp: Tibia 2.3 times longer than patella, cymbium 2.0 times longer than tibia, cymbial apophysis brown and cone-shaped (Figs 25A, 26C); bulb nearly pear-shaped (Figs 25A–B, 26C–D) with papillae proximo-retrolaterally (Figure 25B); spiral ridge brown (Figure 26B), El/Bl ratio 1.81 (Figure 26D), Esl/El ratio 0.44 (Figure 26D).

**Female**: Total length 1.52. Carapace 0.61 long, 0.56 wide. Abdomen 0.89 long, 0.89 wide. Coloration and pattern as in male (Figure 27A–B). Six eyes, well developed, clypeus height 0.12, ocular quadrangle 0.17 wide (Figure 27A). Leg measurements: I 4.35 (1.33, 0.22, 1.39, 0.70, 0.71); II 3.54 (1.08, 0.20, 1.10, 0.63, 0.53); III 2.62 (0.80, 0.19, 0.75, 0.48, 0.40); IV 3.28 (1.03, 0.20, 1.01, 0.61, 0.43). Insemination duct as wide as receptacle (Figure 27C); receptacle U-shaped, slightly swollen at end (Figure 27C).

##### Variation.

In 3♂ paratypes: El/Bl ratio 1.78–1.82, Esl/El ratio 0.43–0.45.

##### Distribution.

China (Guangxi, Figure 31), known only from the type locality.

#### Pinelema zhewang

Taxon classificationAnimaliaAraneaeTelemidae

(Chen & Zhu, 2009) comb. n.

**Figure 28.**
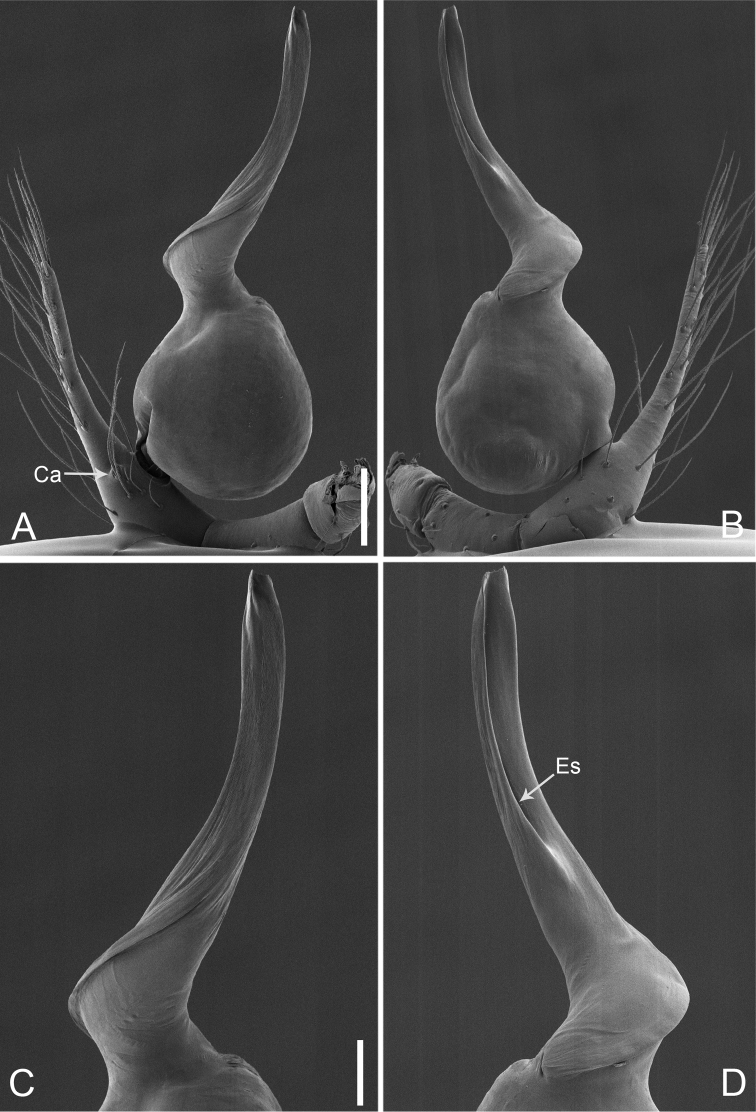
*Pinelemazhewang*, male. **A** Palp, prolateral view **B** Palp, retrolateral view **C** Embolus, prolateral view **D** Embolus, retrolateral view. Scale bars: 0.1 mm (**A–B**), 0.05 mm (**C–D**).

**Figure 29.**
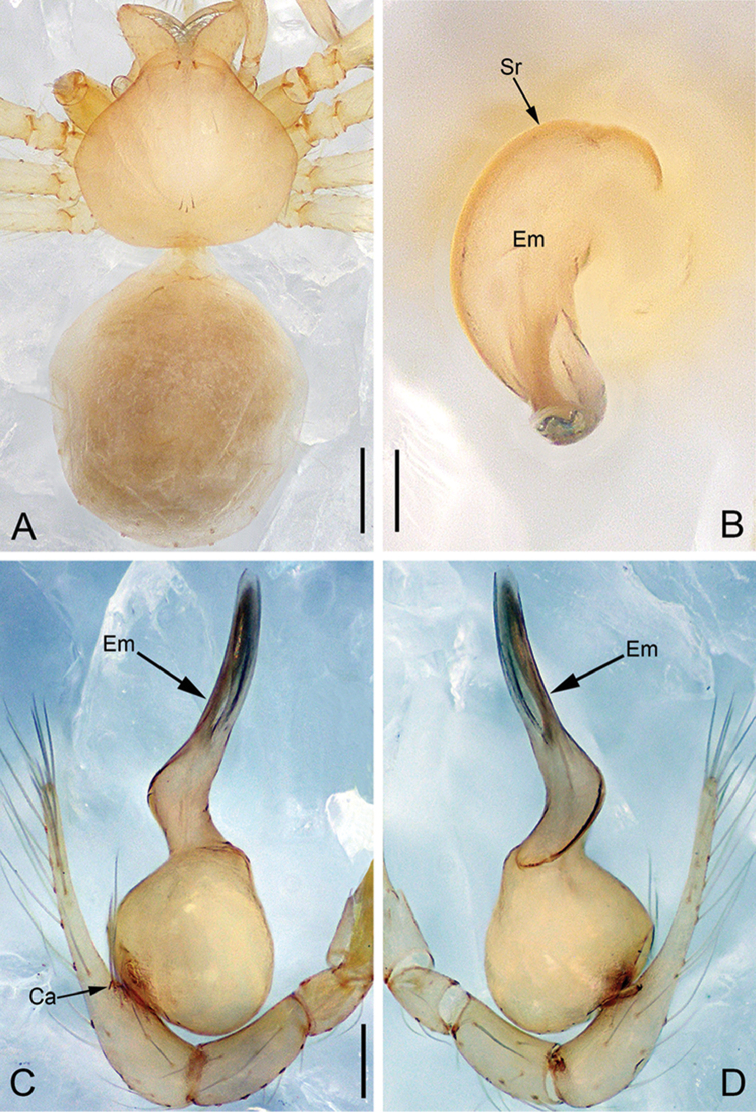
*Pinelemazhewang*, male. **A** Habitus, dorsal view **B** Embolus, apical view **C** Palp, prolateral view **D** Palp, retrolateral view. Scale bars: 0.2 mm (**A**), 0.05 mm (**B**), 0.1 mm (**C–D**).

**Figure 30.**
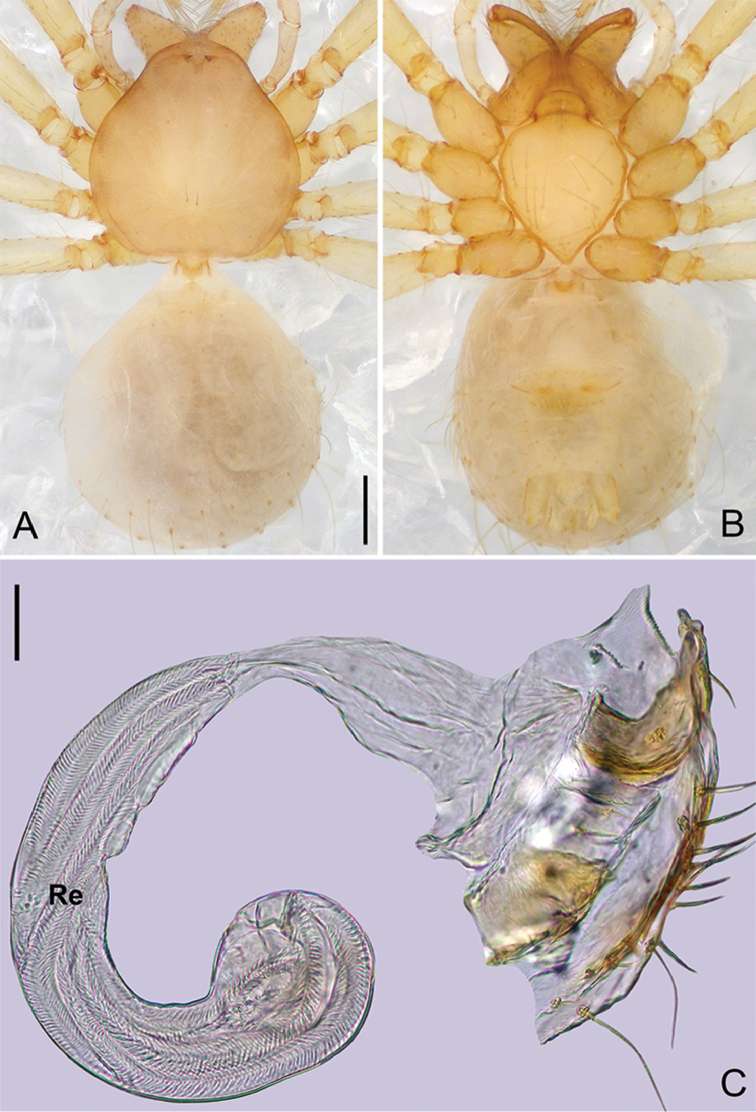
*Pinelemazhewang*, female. **A** Habitus, dorsal view **B** Habitus, ventral view **C** Endogyne, lateral view. Scale bars: 0.2 mm (**A–B**), 0.05 mm (**C**).

**Figure 31.**
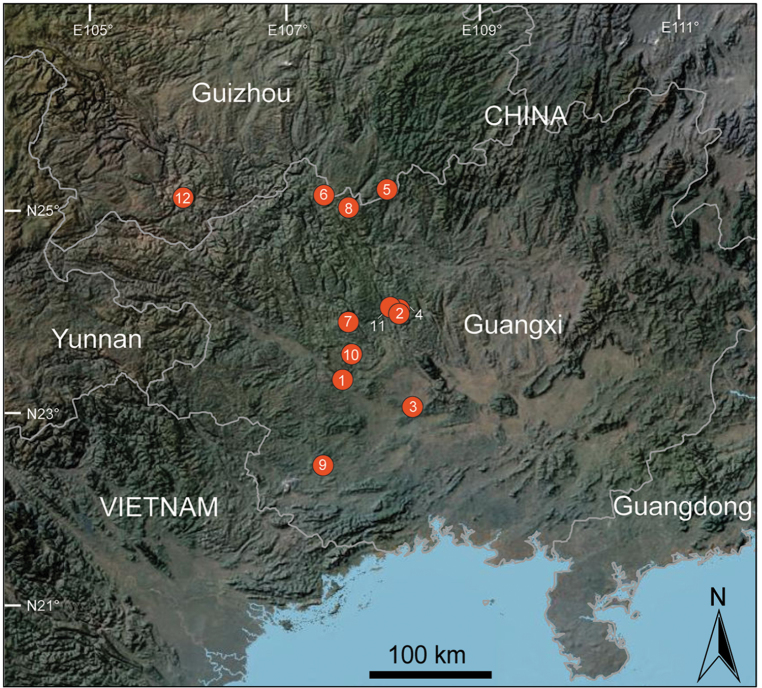
Distribution records of *Pinelemabailongensis* species group: **1***P.bailongensis***2***P.cheni* Zhao et Li, sp. n. **3***P.cordata***4***P.huoyan* Zhao & Li, sp. n. **5***P.liangxi***6***P.lizhuang* Zhao & Li, sp. n. **7***P.strentarsi***8***P.wangshang* Zhao & Li, sp. n. **9***P.wenyang* Zhao & Li, sp. n. **10***P.xiushuiensis***11***P.yunchuni* Zhao & Li, sp. n. **12***P.zhewang*.

Figs 28 , 29 , 30 , 31

Telema zhewang : Chen and Zhu 2009: 1709, figure 3A–D, F–L, O–Q (♂♀).

##### Type material.

Holotype ♂ (MLR): China, Guizhou Province, Qianxinan Prefecture, Ceheng County, Qingping Township, Zhewang village, Zhoujia cave, 600 m, 25°11.000'N, 105°55.000'E, 5.XI.1999, H. Chen and Y. Zhang leg. Paratypes: 13♂ and 22♀ (MHBU), same data as holotype. Not examined.

##### Material examined.

4♂ and 8♀ (molecular vouchers, IZCAS) from the type locality, 10.III.2011, C. Wang and L. Lin leg.

##### Diagnosis.

This species resembles *P.xiushuiensis* (see Figure 24 and Wang and Li 2016: 556, figs 9–12) but can be distinguished by following characters: cymbial apophysis very short (Figs 28A, 29C) (cymbial apophysis long in *P.xiushuiensis*) and larger Esl/El ratio (058–0.63, n = 4, mean: 0.60, Suppl. material 1: Figure S12) (smaller Esl/El ratio 0.50–0.52, n = 5, mean: 0.51 in *P.xiushuiensis*).

##### Description.

Male palp: Embolus bent (Figs 28A–D, 29C–D), spiral ridge brown (Figure 29B), El/Bl ratio 1.73–1.83 (n = 4, mean: 1.76, Suppl. material 1: Figure S12); Esl/El ratio 0.58–0.63 (n = 4, mean: 0.60, Suppl. material 1: Figure S12). Female endogyne: insemination duct as wide as receptacle (Figure 30C); receptacle U-shaped, slightly swollen at end (Figure 30C). For more detailed descriptions, see Chen and Zhu (2009).

##### Comments.

This species shares a combination of morphological characters with *P.bailongensis*, such as the long, tube-shaped embolus (see Figs 28A–D, 29C–D and Chen and Zhu 2009: figure 3C–D), the presence of a cymbial apophysis in the male palp prolaterally (see Figs 28A, 29C and Chen and Zhu 2009: figure 3F), and a U-shaped and medially strongly curved receptacle (see Figure 30C and Chen and Zhu 2009: figure 3J–L), so this species is transferred to *Pinelema*.

##### Distribution.

China (Guizhou, Figure 31), known only from the type locality.

## Supplementary Material

XML Treatment for Pinelema

XML Treatment for Pinelema bailongensis

XML Treatment for Pinelema bailongensis

XML Treatment for Pinelema cheni

XML Treatment for Pinelema cordata

XML Treatment for Pinelema huoyan

XML Treatment for Pinelema liangxi

XML Treatment for Pinelema lizhuang

XML Treatment for Pinelema strentarsi

XML Treatment for Pinelema wangshang

XML Treatment for Pinelema wenyang

XML Treatment for Pinelema xiushuiensis

XML Treatment for Pinelema yunchuni

XML Treatment for Pinelema zhewang

## Supplementary materials

Supplementary material 1 Figures S1–S12. Left male palps of *Pinelemabailongensis* species group, retrolateral view Data type: specimens data This is an open access article distributed under the terms of the Creative Commons Attribution License (CC BY 4.0), which permits unrestricted use, distribution, and reproduction in any medium, provided the original author and source are credited.Huifeng Zhao, Zhiyuan Yao, Yang Song, Shuqiang Li
