# Supplementary material for: Taxonomic study of the Pinelemabailongensis species group with descriptions of six new species from China (Araneae, Telemidae)
Source: Zookeys. 2018 Sep 12;(784):7–57. doi: 10.3897/zookeys.784.27758 (PMC6160845; doi:10.3897/zookeys.784.27758)
Supplement: Supplementary material 1 — Figures S1–S12. Left male palps of Pinelemabailongensis species group, retrolateral view [file zookeys-784-007-s001.docx]

Supplementary - Left male palps of *Pinelema bailongensis* species group, retrolateral view

**Taxonomic study of the *Pinelema bailongensis* species group (Araneae, Telemidae), with descriptions of six new species**

By Huifeng Zhao, Zhiyuan Yao, Yang Song & Shuqiang Li


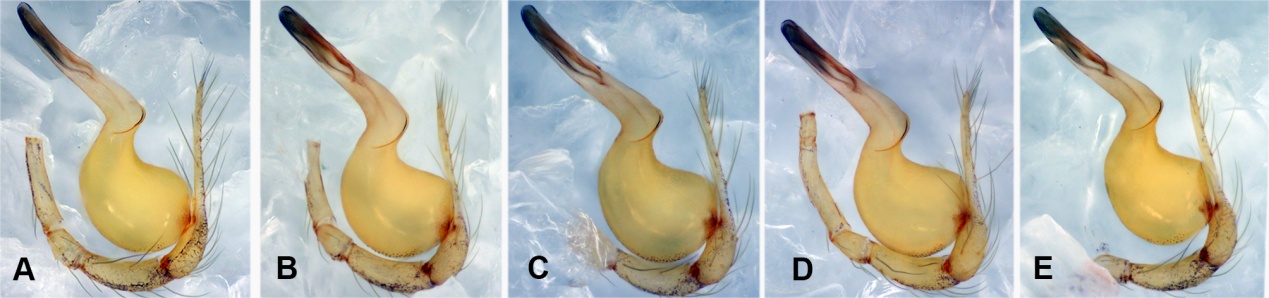


Figure S1. *Pinelema bailongensis* Wang & Li, 2012


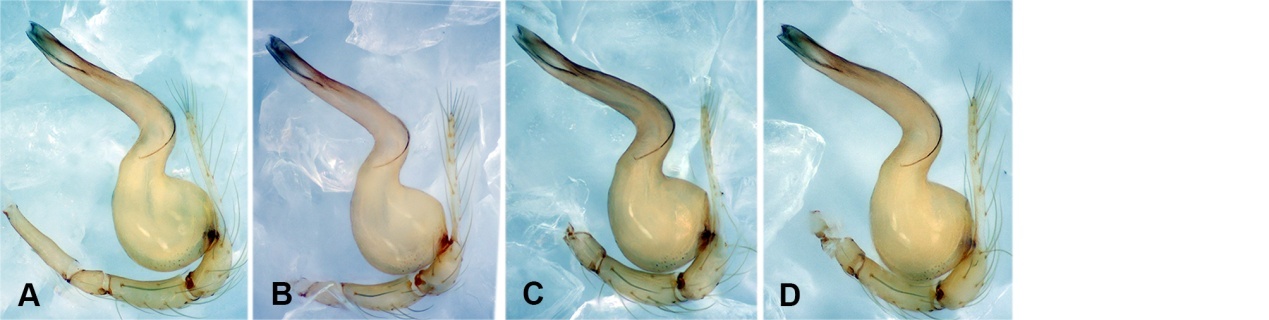


Figure S2. *Pinelema cheni* Zhao & Li sp. n. A: holotye, B–D: paratypes


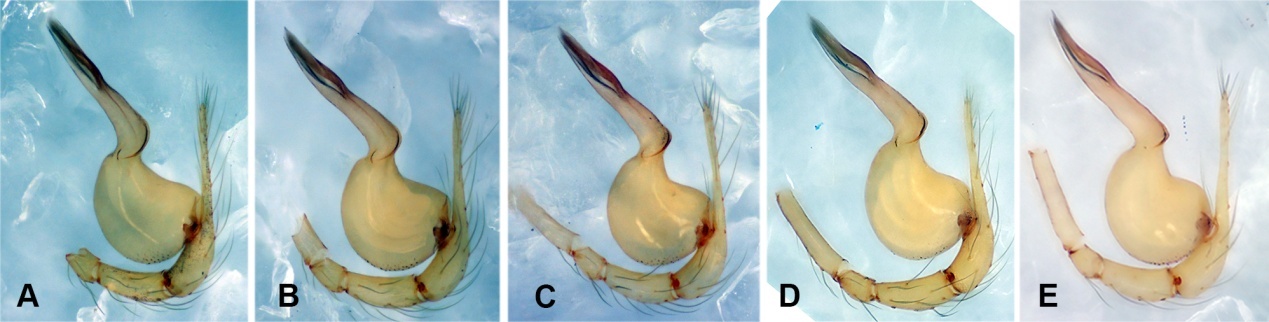


Figure S3. *Pinelema cordata* (Wang & Li, 2010) comb. n.


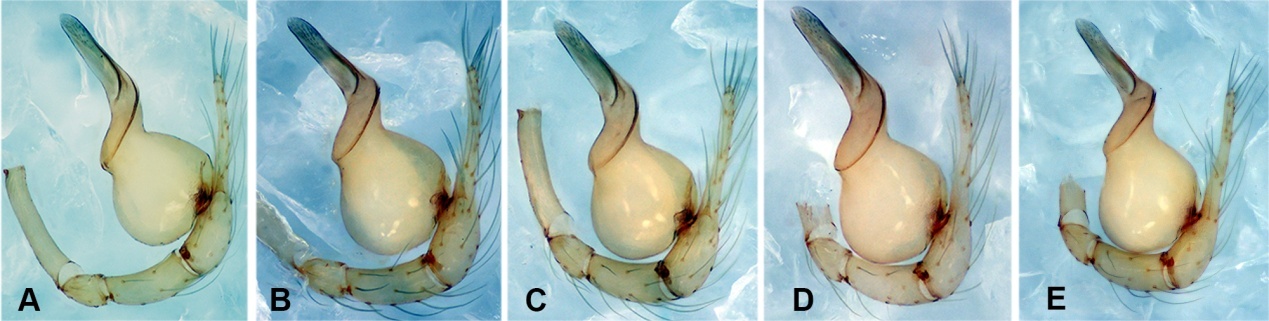


Figure S4. *Pinelema huoyan* Zhao & Li sp. n.


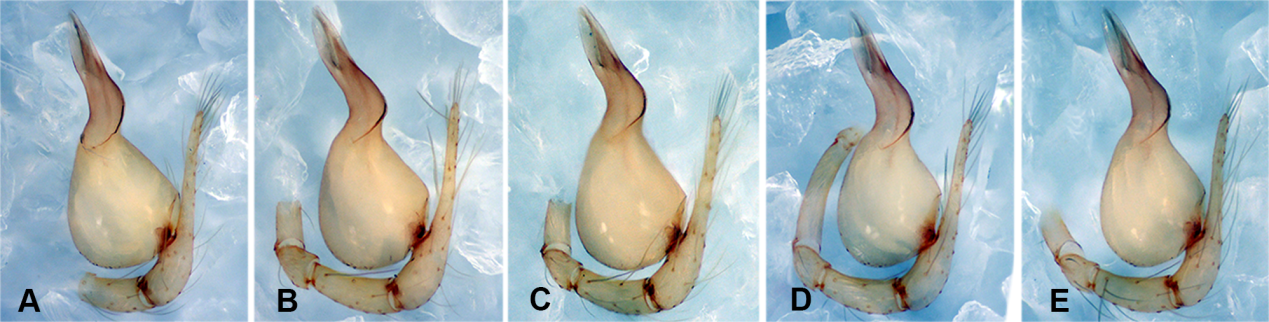


Figure S5. *Pinelema liangxi* (Zhu & Chen, 2002) comb. n.


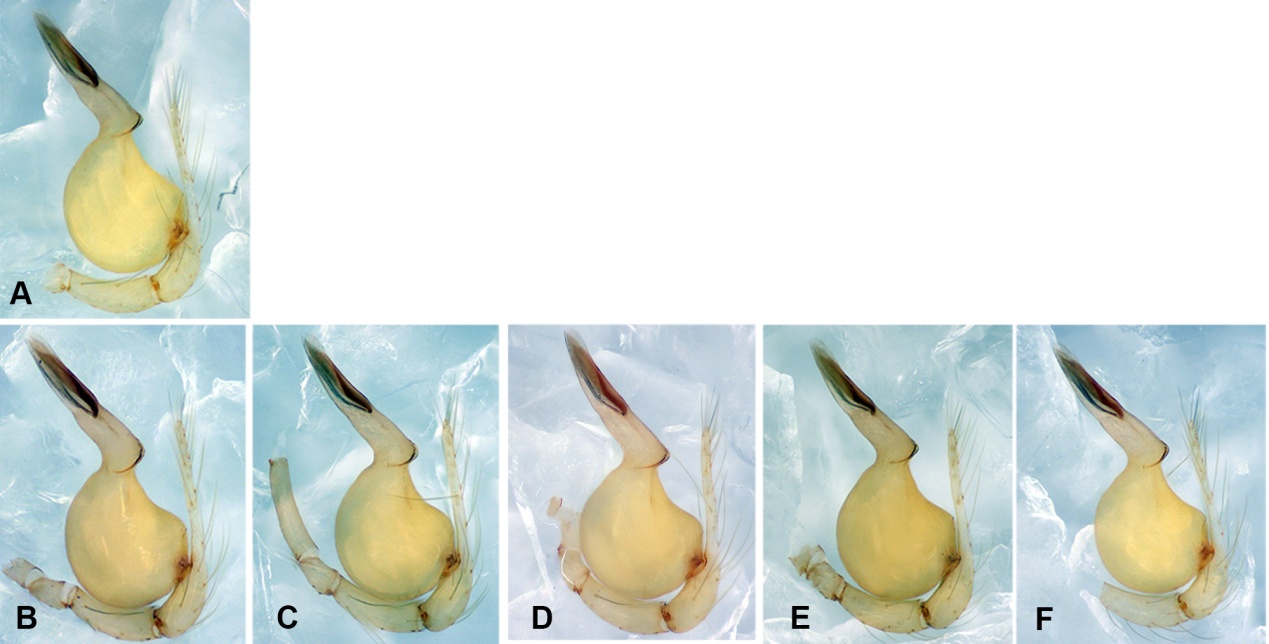


Figure S6. *Pinelema lizhuang* Zhao & Li sp. n. A: holotye, B–F: paratypes


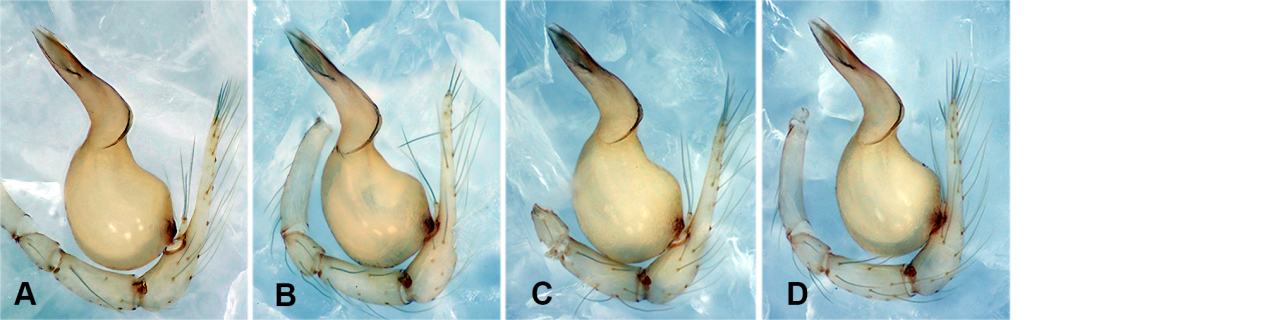


Figure S7. *Pinelema strentarsi* (Lin & Li, 2010) comb. n.


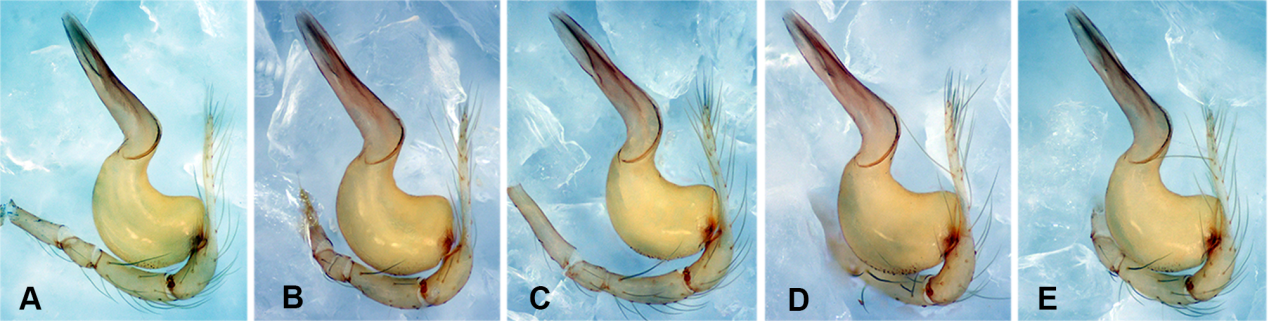


Figure S8. *Pinelema wangshang* Zhao & Li sp. n. A: holotye, B–E: paratypes


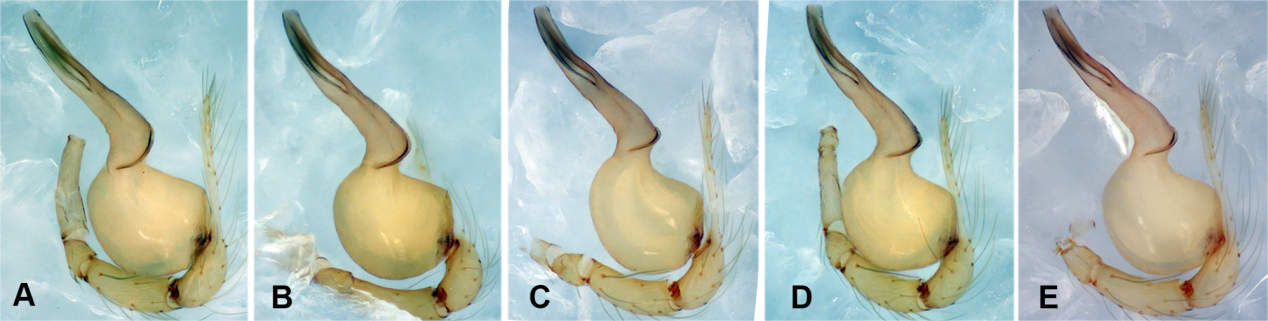


Figure S9. *Pinelema wenyang* Zhao & Li sp. n. A: holotye, B–E: paratypes


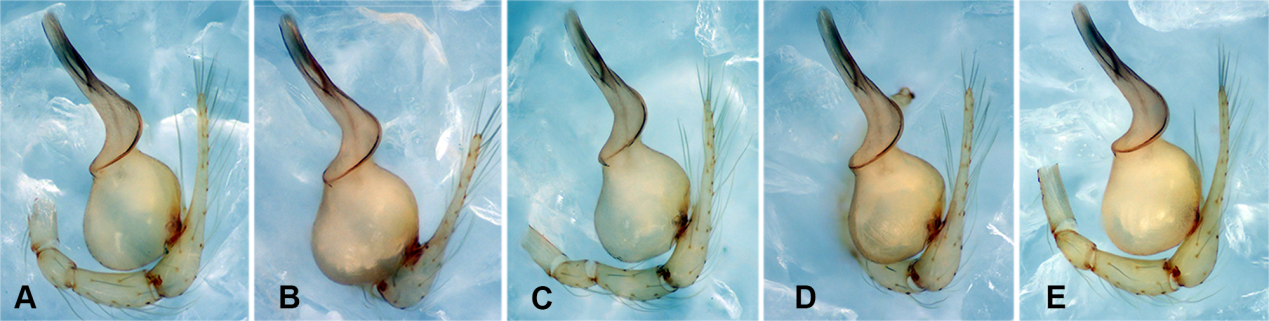


Figure S10. *Pinelema xiushuiensis* Wang & Li, 2016.


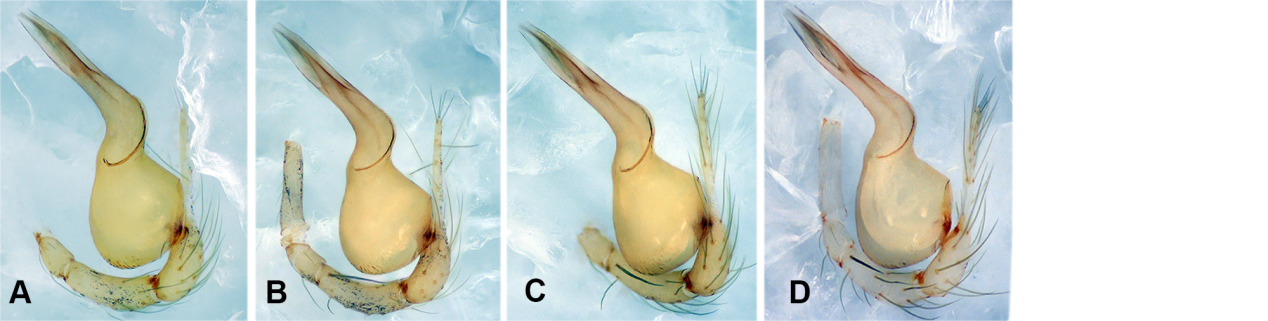


Figure S11. *Pinelema yunchuni* Zhao & Li sp. n. A: holotye, B–D: paratypes


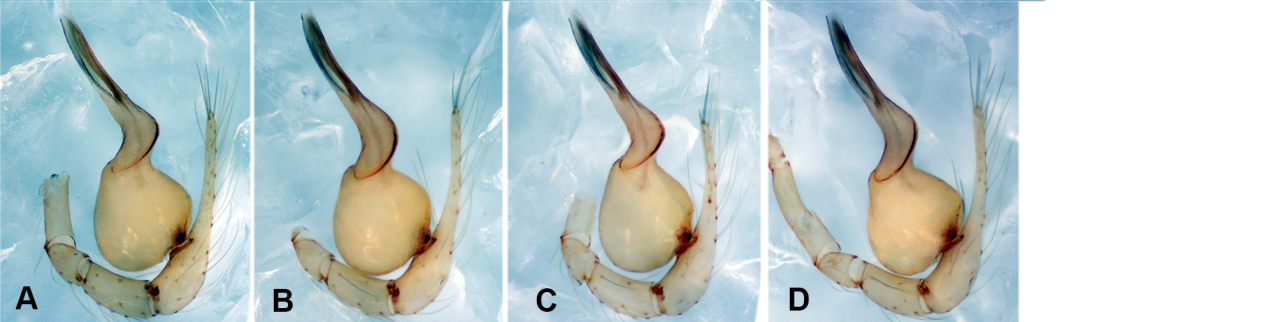


Figure S12. *Pinelema zhewang* (Chen & Zhu, 2009) comb. n.
